# The epigenetic mechanisms of adaption to the hot and humid climate in Hu sheep (*Ovis aries*)

**DOI:** 10.14814/phy2.16164

**Published:** 2024-12-26

**Authors:** Hangxing Ren, Jing Jiang, Jie Li, Xiaoyan Sun, Cancan Chen, Liangjia Liu, Shipeng Lv, Minghao Qu, Yuxue Fan, Peng Zhou, Gaofu Wang

**Affiliations:** ^1^ Chongqing Academy of Animal Sciences Chongqing China; ^2^ Chongqing Engineering Research Center for Goats Chongqing China

**Keywords:** adaptation, epigenetics, hot and humid climate, sheep

## Abstract

Hu sheep is characterized by its excellent fecundity and high adaptability to various ecological environments. To reveal the molecular basis involved in Hu sheep, we first examined the 10 index of neuroendocrine and metabolism in blood in Hu sheep during non‐stress period (April–May) and stress period (July–August) using ELISA, including CRH, adrenocorticotropic hormone (ACTH), cortisol, aldosterone, adrenaline, T3,T4, SOD, GSH‐PX, and T‐AOC. Then we conducted the Whole genome DNA methylation sequencing in blood and performed the comparative analysis of global DNA methylation between the non‐stress period and the stress period. Our results demonstrated that among the 10 index tested in blood, only ACTH, T3, and T‐AOC were significantly changed (*p* < 0.01) in Hu sheep between two periods. This indicates Hu sheep's special adaptability to the high hygrothermal environment takes the decrease of metabolic level and total antioxidant capacity as compensation, which differ obviously from other intolerant hygrothermal animals. At the epigenetic level, differential methylation of TPO, ADCY9, PRKACB, and CREB5 play important roles in excellent resistance to hygrothermel environment in Hu sheep by modulation of the secretion of in neuroendocrine hormones (T3, ACTH) and thermogenesis. These findings are valuable for breeding the novel animal breeds resistant to climate stress.

## 1 INTRODUCTION

It is known that the response of organisms to external environment is mainly realized by epigenetic modifications. DNA methylation is the first epigenetic modification ever found, which exists widely in the whole genome and changes dynamically with the change of cell state. The variation of DNA methylation caused by environmental changes also makes genetically homogeneous individuals with different phenotypes (Gao et al., 2010). For example, targeted mutation of the DNA methylation pathway in the Jin algae reduced the genomic methylation level and changed the adaptability of Jin algae to heat, cold, salt and waterlogging (Boyko et al., 2010). DNA methylation also plays important roles throughout the life‐time of animals, such as embryonic development and cell differentiation (Lei et al., 1996; Li et al., 1992; Okano et al., 1999), aging (Ghinassi et al., 2023; Vaidya et al., 2023), and disease (Richardson, 2002). Recent investigations in sheep (*Ovis aries*) demonstrate that DNA methylation affect a variety of physiological status including development, growth, estrus, and pregnancy (Braz et al., 2022; Cao et al., 2023; Capra et al., 2017; Di et al., 2023; Fonseca et al., 2023; Guo et al., 2020; He et al., 2018; Narayan et al., 2022). Based on the study of ancient DNA samples of bison, it is found that the adaptation of animals to the rapidly changing climate environment in the last glacial period mainly depends on epigenetics, and rarely involves the change of DNA sequence (Llamas et al., 2012). These indicate that DNA methylation is a conserved mechanism by which animals and plants respond to environmental stimuli.

The neuroendocrine stress system is largely under control of the hypothalamus. Both physical and psychological stressors activate the hypothalamic–pituitary–adrenal (HPA) axis by rapidly triggering the release of the corticotropin releasing hormone (CRH) from the paraventricular nucleus (PVN), which in turn causes the secretion of adrenocorticotropic hormone (ACTH) from the pituitary into the blood (de Kloet et al., 2005). ACTH stimulates the synthesis and secretion of glucocorticoids (GCs) from the adrenal cortex, that is, corticosterone (CORT) and cortisol in rodents and humans, respectively. The HPA axis is under negative feedback control of GCs binding to glucocorticoid receptors (GRs) in the hypothalamus and pituitary gland, leading to the inhibition of CRH and ACTH release and causing tight regulation of the stress response system (Yam et al., 2015). Therefore, the level of neuroendocrine hormones in blood is often used as the indicator of evaluation of stress in animals.

Hu sheep is a famous indigenous breed in China, characterized by its all‐year‐around estrus, multiple lambs per litter, and high adaptability to various ecological environments. Recent studies have demonstrated that the Hu sheep can well accommodate the heat and humidity climate (Zhou et al., 2016; Wang et al., 2018). Therefore, epigenetics, especially in DNA methylation must play important roles in reponse to this extremely humid and hot climate environment in Hu sheep. Previous study have investigated the physiology of liver and hypothalamus in Hu sheep, and identified some important genes and pathways involved in response to heat stress (Li et al., 2019a; Li, Kong, et al., 2019b; Lu et al., 2021; Lu, Chu, et al., 2019a; Lu, Ma, et al., 2019b; Silva et al., 2019), which contribute to our understanding of the molecular mechanisms of adaptability to environment in Hu sheep. However, it is yet unknown how would Hu sheep respond to the environment stimuli at blood neuroendocrine level under the condition of two environmental factors (humidity and temperature). In the present study, we examined the blood neuroendocrine hormones associated with environment stress and whole genome DNA methylation in Hu sheep under two different climate conditions to reveal the mechanisms of its excellent resistance to the hygrothermal environment. Our study is not only important to reveal the molecular mechanisms of adaptation to the climate of high humidity and high temperature in ruminant, but also can provide the basic and valuable evidence for breeding the novel animal breeds resistant to the humidity and heat stress in future.

## 2 MATERIALS AND METHODS

### 2.1 Experimental design

According to the characteristics of local climate in Rongchang District, Chongqing, China, we designed the period of April as the non‐stress stage for Hu sheep, during which the average temperature maintained 22.6°C ± 2.58, the average relative humidity was 64 ± 3.05 (×100%). On the other hand, we designed the period of August as the stress stage for Hu sheep, during which the average temperature maintained 33.4°C ± 2.80, the average relative humidity was 85 ± 2.17 (×100%). Here we use the formula developed by NRC (1971) to calculate temperature humidity index (THI) for estimation of the climate stress at two stages in Hu sheep. THI = (1.8 × AT_avg_ + 32) − [(0.55–0.0055 × RH_avg_) × (1.8 × AT_avg_ − 26)], where AT_avg_ is the average AT (ambient temperature) (°C) and RH_avg_ is mean RH (Relative Humidity) (%) (National Research Council, 1971). Twenty heathy 2–3 years Hu ewes were chosen (bodyweight 37 ± 1.69 kg) from the farm of Rongcheng Gouyang Co., Ltd in Chongqing Province. Peripheral venous blood of each ewe was collected for examination of neuroendocrine hormones and DNA methylation of genome at the non‐stress stage and the stress stage, respectively. The serums were isolated for testing the blood hormone level in each ewe at two stages.

### 2.2 Examination of blood neuroendocrine and metabolic hormones

Blood was collected from the jugular vein of the experimental sheep and placed in a 10 mL centrifuge tube for 30 min, then centrifuged at 3000 *r*/min at 4°C for 10 min and stored at −20°C for testing the level of blood neuroendocrine and metabolic hormones. In the present study, we examined the 10 indexes of neuroendocrine in serum in Hu sheep during non‐stress period (*n* = 18) and stress period (*n* = 11), including (CRH, MM‐2560O2, Jiangsu Enzyme Exemption Industry Co., Ltd), adrenocorticotropic hormone (ACTH, MM‐2559O2, Jiangsu Enzyme Exemption Industry Co., Ltd), cortisol, aldosterone, epinephrine, thyroid hormone (T3, MM‐2572O2, Jiangsu Enzyme Exemption Industry Co., Ltd; T4, MM‐2585O2, Jiangsu Enzyme Exemption Industry Co., Ltd), superoxide dismutase (SOD, MM‐1815O2, Jiangsu Enzyme Exemption Industry Co., Ltd), glutathione peroxidase (GSH‐PX, MM‐1817O2, Jiangsu Enzyme Exemption Industry Co., Ltd), and total antioxidant capacity (T‐AOC, ADS‐W‐KY005‐48, Jiangsu Aidi Biotechnology Co., Ltd), using ELISA kits according to the manufacturer's protocol. The differences in the hormone level were measured by T‐test method between two stages. *p* < 0.05 indicates the significant difference in an index between two stages.

### 2.3 Whole genome DNA methylation sequencing

The genome DNA was extracted from blood of three ewes at two stages using DNA extraction kit (13,343, Qiagen), respectively. Whole genome bisulfite sequencing (WGBS) for the sheep whole genome DNA were performed by the Wuhan Frasergen Information Co., Ltd (China). Simply, Adapters and low quality (phred quality <10) bases were removed from raw seqenceing reads with Trimmomatic (version 0.38) and them trimmed reads were aligned to reference genome with Bismark (version 0.22.3). PCR duplications were removed with tools from Bismark and then organelle contaminations were excluded from downstream analysis with custom scripts. Bisulfite convertion rate was estimated with reads from lambda bacteriaphage. In briefing, methylated, as well as unmethylated cytosines calls from all context (CG, CHG, and CHH) were used to determine the convertion rate. We defined the bisulfite convertion rate (BCR in short) as BCR = unmethylated cytosine calls/methylated cytosine calls. A BCR value higher than 99% was considered as successful bisulfite treatment.

Data from both strand were merged for CG context and a minimum coverage of 5 for each CG site were set to exclude potential methylation level bias in further analysis.

R package “bsseq” and “dmrseq” were used to identify DMRs (differentially methylated regions). A valid DMR requires *Q*‐value not larger than 0.05, plus at least 20% difference in methylation level between two groups and at least 10 CG sites in it. DMRs were assigned to nearest TSS using R pckage “ChIPseeker” and gene function enrichment analysis were conducted with R package “ClusterProfiler”.

## 3 RESULTS

### 3.1 Effects on physiological status in neuroendocrine and metabolism

**FIGURE 1:**
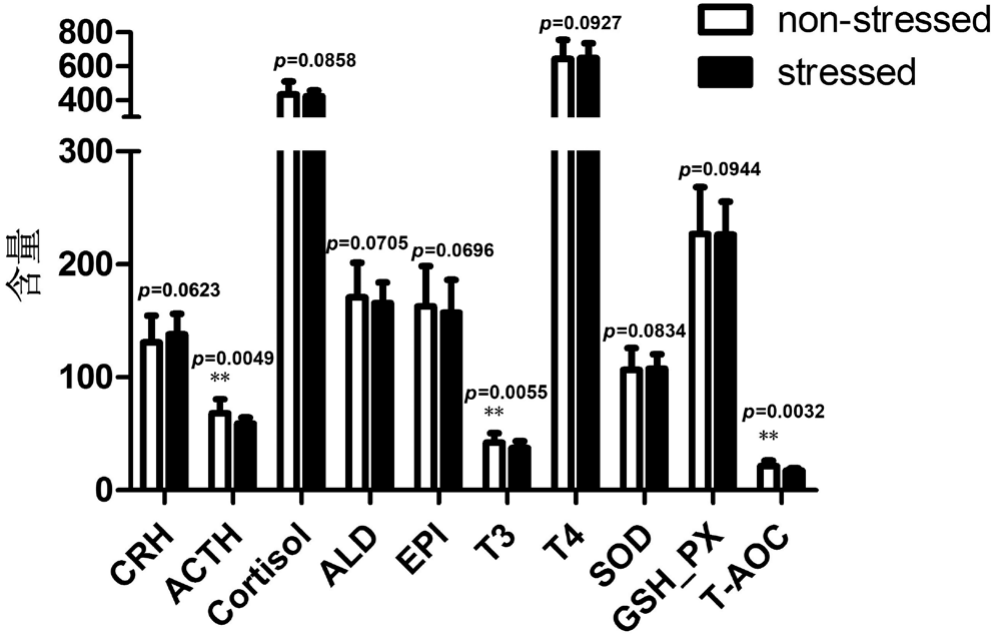
The neuroendocrine and metabolism indexes in serum in Hu sheep at non‐stress and stress stages. Data are presented as mean ± SEM of all samples at individual stage. **Indicates significant difference at level of 0.01 (*p* < 0.01).

The THI were calculated to represent the stress level of Hu sheep at two stages. Our results illustrated that THI is 69.73 and 89.31 at the non‐stress stage (April) and stress stage (August), respectively. To evaluate the effects of humidity and heat stress on neuroendocrine and metabolism in Hu sheep, we examined the levels of 10 blood indexes (CRH, ACTH, cortisol, ALD, EPI, T3, T4, SOD, GSH‐Px, and T‐AOC) in ewes under the non‐stress (April) and stress conditions (August). Our results demonstrated that the levels of blood ACTH, T3, and T‐AOC in ewes at stress stage were significantly lower than those at non‐stress stages (*p* < 0.01). There were no significant differences in other seven blood indexes of ewes at two stages (Figure 1). Since ACTH and T3 are important hormones involved in metabolism of sugar, protein and fat and thus providing energy for the body of animals.

### 3.2 Genome‐wide DNA methylation in blood in Hu sheep

To investigate the epigenetic mechanism of the resistance to humidity and heat stress in Hu sheep, we examined the methylation status of genome DNA of ewes (*n* = 3) under the non‐stress and stress conditions. We obtained a total of 1,414,248,466 reads in six samples for subsequent identification of the differential methylated regions (DMRs) at two stages (File S1). The variety of average methylation levels in the whole genome (File S2) and the sequencing depth of genome‐wide CG loci in each sample (File S3) were also calculated. Results illustrate that the mCG is the major form of methylation in sheep genome, whose percentage is 72.19%–73.61% in the six samples. The above analyses indicate that the WGBS–seq data is qualified for further analysis.

**FIGURE 2:**
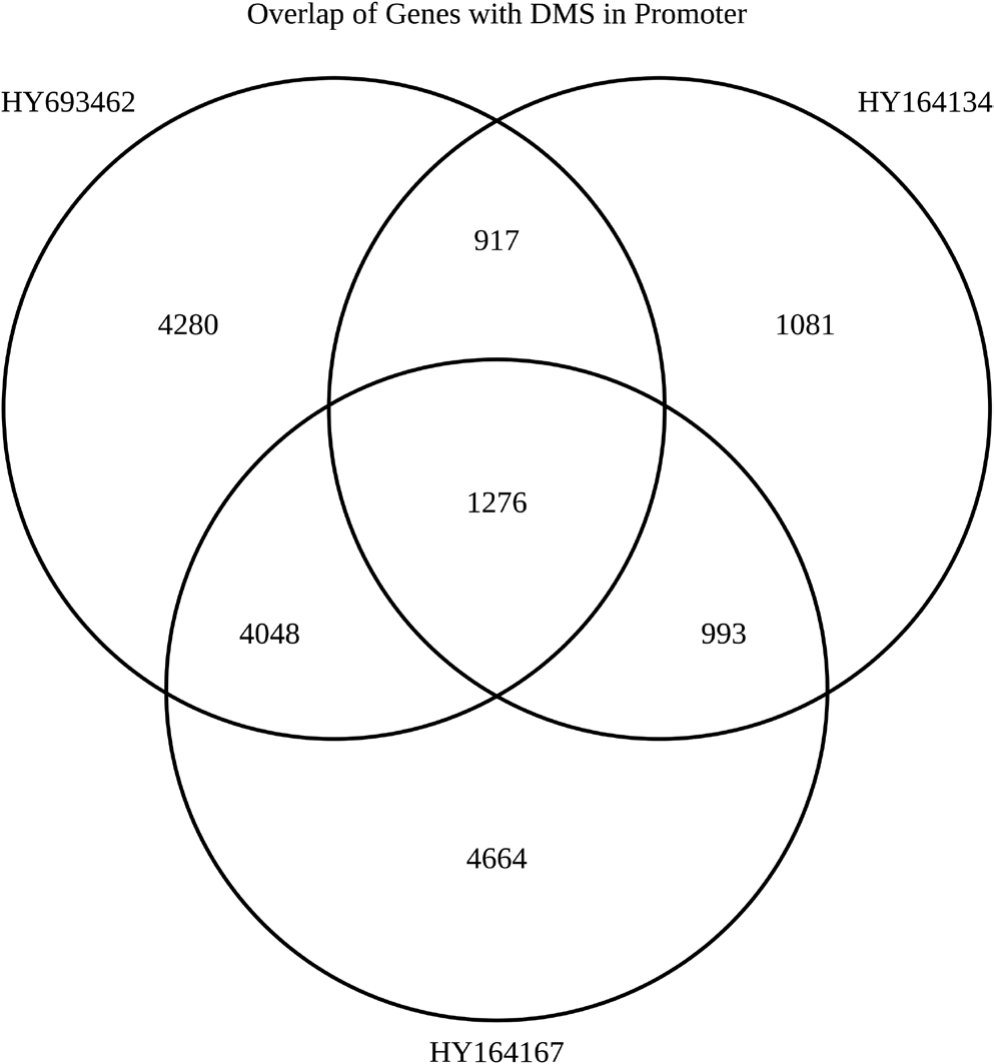
Venn diagram of the genes with differentially methylated site (DMS) in three samples of Hu sheep at two stages.

**FIGURE 3:**
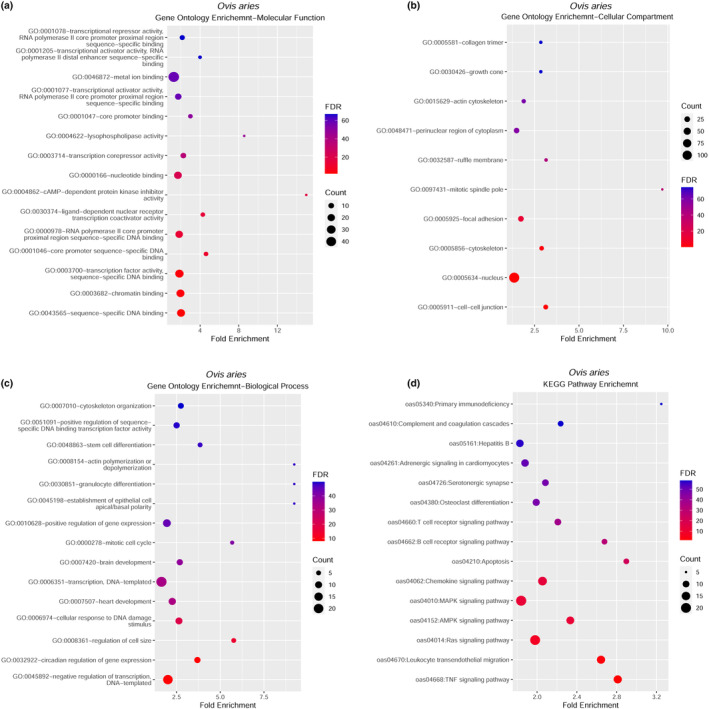
Enrichment analysis of GO terms (Molecular function (a), Cellular compartment (b), Biological progress (c)) and KEGG (d) in DMS of Hu sheep at two environmental conditions.

To identified the differentially methylated regions (DMRs), we examined the distribution of global methylation level in two groups (unstressed vs. stressed) (File S4) as well as estimated the correlation among samples (File S5). Unexpectedly, we did not find any significant DMRs in sheep genome between two stages (groups) as given analytic criteria (File S6). Subsequently, the alternative plan was employed to identify the candidate DMRs involved as described below. First, we identified the DMRs in each of three individuals between two stages, respectively. Then we extracted the 1267 DMRs in common among three individuals to subsequent functional annotation and enrichment analysis (Figure 2). Our results demonstrated that these differential methylated (DM) genes were significantly overrepresented in GO (Gene Ontology) terms and KEGG pathways (Figure 3). As to molecular function, they were involved in sequence‐specific DNA binding (GO:0043565), chromatin binding (GO:0003682), transcription factor activity sequence‐specific DNA binding (GO:0003700), RNA polymerase II core promoter proximal region sequence‐specific DNA binding (GO:0000978), and core promoter sequence‐specific DNA binding (GO:0001046), etc. (Figure 3a). As to cellular compartment, most of the DM genes were enriched in nucleus (GO:0005634) and focal adhesion (GO:0005929) (Figure 3b). As to biological progress, they were associated with negative regulation of transcription, DNA–template (GO:0045892), circadian regulation of gene expression (GO:0032922), regulation of cell size (GO:0008361), and cellular response to DNA damage stimulus (GO:0006974) (Figure 3c). For KEGG pathways, the DM genes were significantly enriched in TNF signaling pathway (oas04668), Leukocyte transendothelial migration (oas04670), Ras signaling pathway (oas04014), AMPK signaling pathway (oas04152), MAPK signaling pathway (oas04010), and so on (Figure 3d). This indicates that these GO terms and KEGG pathways probably participate in the process of adaptation to the humidity and heat environment in Hu sheep.

### 3.3 Key gene methylation related to adaptability to humidity and heat stress in Hu sheep

**FIGURE 4:**
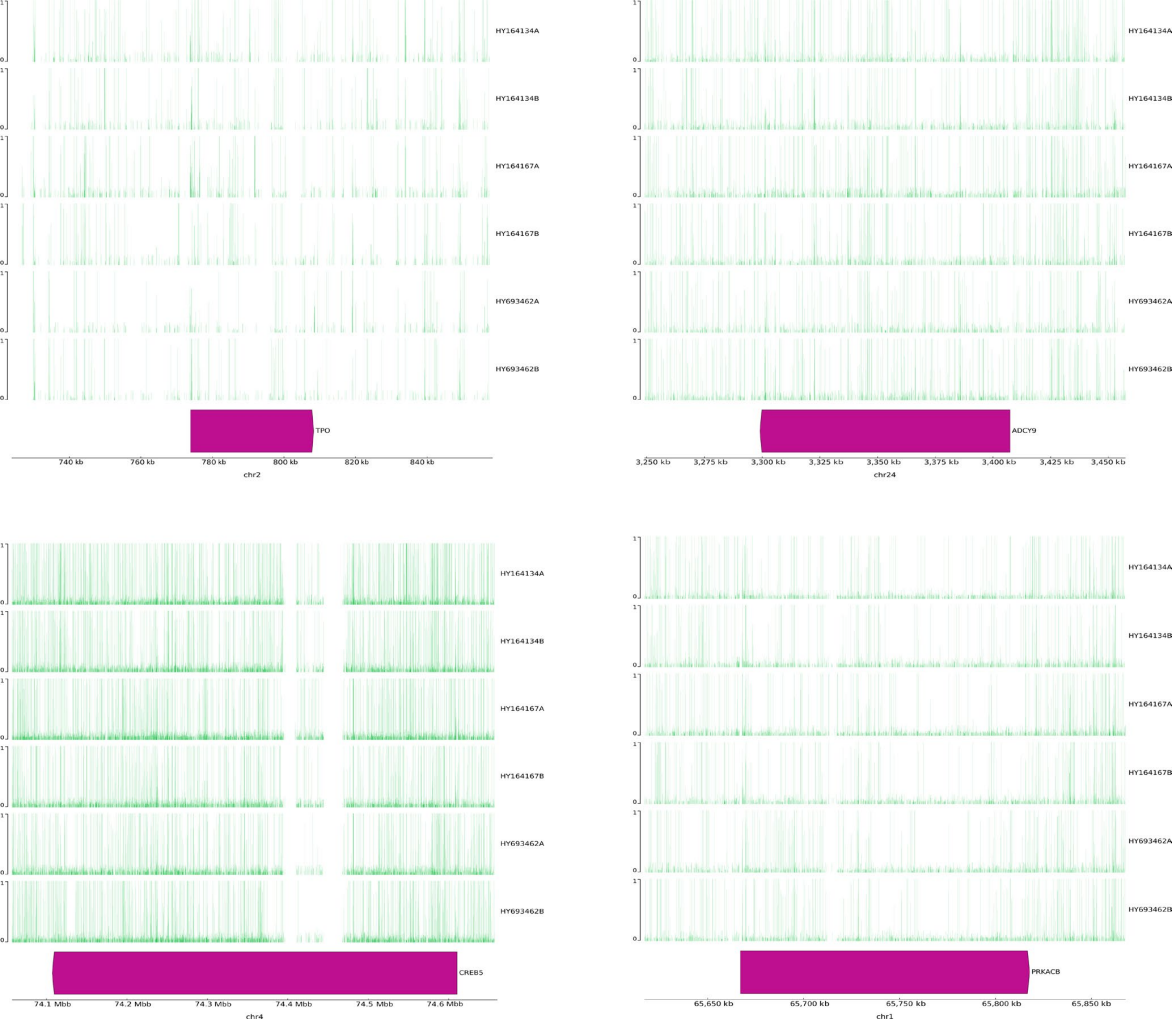
Visualization of the methylation level in *TPO*, *ADCY9*, *PRKACB*, and *CREB5* gene at non‐stress (a) and stress stages (b) in Hu sheep. The gene bodies are indicated as rose red bars on individual chromosome in sheep genome, whose arrow indicate their five points (namely TSS–Transcription Star Site). The methylation degree of each region is illustrated by the green vertical line on the gene body with the range of 0–1.

**FIGURE 5:**
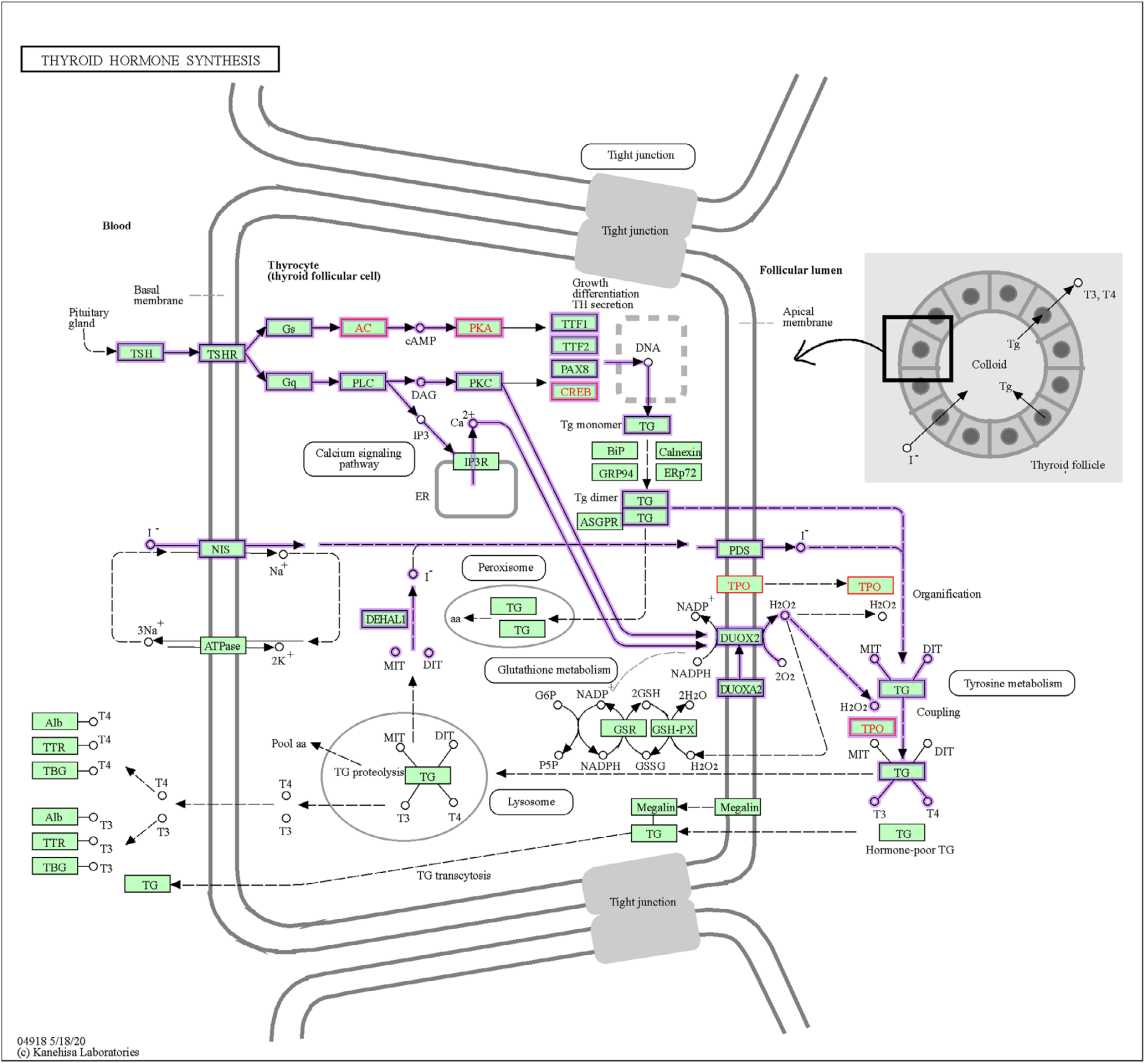
Thyroid hormone synthesis pathway and network of TRH‐TSH‐TH signaling in Hu sheep. The Hu sheep's differentially methylated genes (red fonts) between two stages were overrepresented in *Thyroid hormone synthesis* pathway (hsa04918) in KEGG database, which contains the network of TRH‐TSH‐TH signaling (nt06322) (purple lines).

**FIGURE 6:**
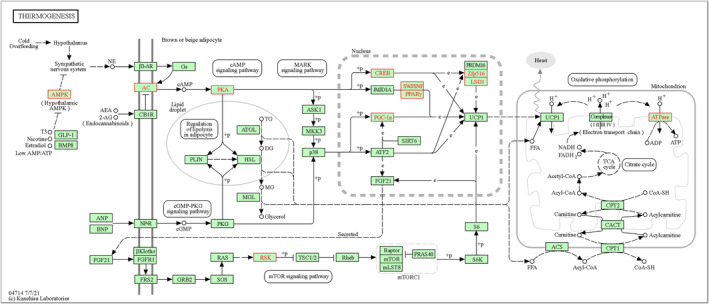
Regulation of thermogenesis in Hu sheep. The Hu sheep's differentially methylated genes (red fonts) between two stages were overrepresented in *Thermogenesis* pathway (hsa04714) in KEGG database.

**FIGURE 7:**
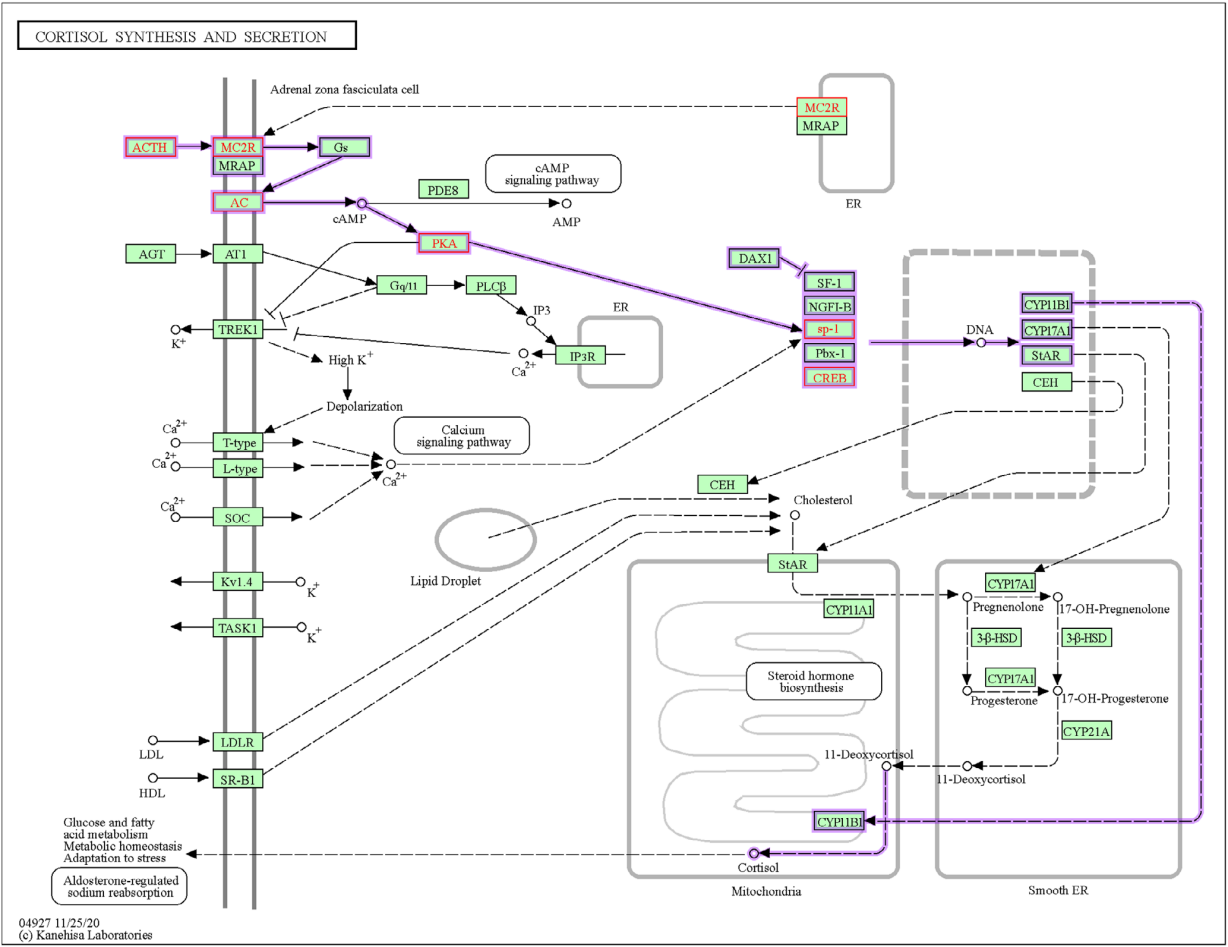
Cortisol synthesis and secretion pathway and network of CRH‐ACTH‐cortisol signaling in Hu sheep. The Hu sheep's differentially methylated genes (red fonts) between two stages were overrepresented in *Cortisol synthesis and secretion* pathway (hsa04927) in KEGG database, which contains the network of CRH‐ACTH‐cortisol signaling (nt06310) (purple lines).

To further explore the potential key pathways or genes involved in the resistance to humidity and heat stress in Hu sheep, we combined the hormonal index of neuroendocrine and metabolism (Figure 1) and the DMRs between two environmental conditions. In the present study, we focused on the metabolism pathways related to T3 and ACTH in KEGG database and found the Thyroid hormone synthesis, Thermogenesis, and CRH‐ACTH‐cortisol signaling pathways were closely associated with the adaptive regulation of Hu sheep under environmental stress. Especially, some DM node genes may play crucial roles in regulating these signals, such as *TPO*, *ADCY9*, *PRKACB*, and *CREB5* (Figure 4, Table 1). For example, *TPO*, the protein of which acts as a key enzyme in generation of the thyroid hormones, thyroxine and triiodothyronine (T3) (Figure 5). The repression of *TPO* transcription mediated by methylation compromised the synthesis of T3, which would further result in the reduction of heat production through Thermogenesis pathway (Figure 6). In addition, methylation of six genes in the CRH‐ACTH‐cortisol signaling pathway would greatly contribute to inhibit the secretion of blood cortisol in Hu sheep under the stress stage (Figure 7). The methylation of these key genes provided further evidence that Hu sheep take the decrease of metabolic rate and total antioxidant capacity as a compensation to accommodate the climate of high humidity and high temperature in Chongqing, China.

**TABLE 1:** The signaling pathways associated with the adaption to hygrothermal environment in Hu sheep.

ID	KEGG pathway	Differentially methylated gene	*p*‐Value	Corrected *p*‐value
hsa04918	Thyroid hormone synthesis	*ADCY9, PRKACB, CREB5, TPO*	1.44E‐11	1.20E‐09
hsa04714	Thermogenesis	*ADCY9, PRKACB, CREB5, PPARGC1A, PRKAG2, PPARG, SMARCA2, KDM1A, ZNF516, ATP5MF, RPS6KA2*	3.89E‐25	3.69E‐23
hsa04927	CRH‐ACTH‐cortisol signaling	*POMC, MC2R, ADCY9, PRKACB, CREB5, SP1*	2.82E‐17	2.48E‐15

## 4 DISCUSSION

According to the standard of criterion, animals have a stress response under the environmental THI > 72. Heat stress hinders animals' normal physiological metabolisms and performances in husbandry production. Yan & Li (2011) showed that the relative humidity increased from 57% to 78% at a temperature of 35°C, and body temperature of rams increased by 0.6°C (Yan & Li, 2011), suggesting the interaction between temperature and relative humidity can aggravate their adverse effects on animals, which were confirmed by recent study on sheep and goats (Can et al., 2017; Li et al., 2018). As a characteristic breed of sheep in China, Hu sheep are well able to adapt to the high damp heat condition, which endows with it significant economic value in livestock production. In the present study, we found that the pattern of blood hormones in Hu sheep was obviously different from other intolerant animals who had greater plasma cortisol level under the similar stress conditions (Zhang et al., 2017; Chen et al., 2018; Li et al., 2018; Wang et al., 2010; Yadav et al., 2015). Our results indicate that Hu sheep seem to adopt the alternative endocrine pathways to acclimate the damp heat environment, namely they take the decrease of metabolic rate and total antioxidant capacity as a compensation. It is known that the T3 signaling pathway plays vital roles in regulation of lipid peroxidation and antioxidant enzyme activities (Chu et al., 1995; Sreejith & Oommen, 2006; Varghese et al., 2001; Wrutniak et al., 1998). The downregulation of T3 level in blood of Hu sheep may explain their decrease of T‐AOC in blood at the stress stage in the present study (Figure 1). In addition, decreasing of the ATCH hormone contributes to remain a relatively constant level of blood cortisol in Hu sheep under the high damp heat condition contrast to the non stressed stage through the *CRH‐ACTH‐cortisol signaling* pathway (Figure 7) (discussed below).

TPO, as a crucial enzyme of synthesis of thyroid, mutation of which caused the clinical hypothyroidism or dishormonogenesis in human (Balmiki et al., 2014; Cangul et al., 2013; Pfarr et al., 2006; Tenenbaum‐Rakover et al., 2007; Turkkahraman et al., 2009). In the present study, methylation in *TPO* gene suggested the repression of *TPO* transcription and thus compromising the synthesis of T3 by *Thyroid hormone synthesis* pathway (Figure 5), thus resulting in reduction of thermogenesis under hygrothermal stress in Hu sheep (Figure 6). Most importantly, we found an interesting fact that the level of plasma cortisol in blood remained stable in Hu sheep under the stress situation as compared to the control, suggesting that there is a special mechanism underpinning their excellent adaptability to the damp heat environment. The stress induced promoter methylation of *POMC*, *MC2R*, *ADCY9*, *PRKACB*, *CREB5*, and *SP1* clearly suggested the resulting inhibition of secretion of the cortisol through the *CRH‐ACTH‐cortisol signaling* pathway (Figure 7), which is obviously different from other animals with increasing cortisol level in blood under heat stress (Bagath et al., 2019; Gong et al., 2015; Hooper et al., 2018; Megahed et al., 2008; Oluwagbenga et al., 2022; Rasooli et al., 2010; Wang et al., 2015). Therefore, the *CRH‐ACTH‐cortisol signaling* pathway could be a breakthrough to uncover the mystery of this excellent trait in Hu sheep. Moreover, a most recent study also showed that common introgression signals of alleles *ADCY3*, *TRPV1*, and *PADI2* genes from wild relatives enhanced climatic adaptation and resistance to pneumonia in domestic sheep (National Research Council, 1971). This implies the important roles of *ADCY* family in adaptation to environments in sheep.

In general, adaptability is a physiological process involved in multiple organs and systems, which is dynamic and hormone dependent by affecting elaborate networks of genes and global patterns of gene expression. It is triggered by epigenetic changes such as DNA methylation through modulation of the regulatory triad (DNA binding, chromatin modulation, and transcriptional activation). As described by Evans & Mangelsdorf (2014), physiologic complexity must arise from genomic complexity reflected by a coordinated genome‐wide interaction of the receptors with DNA (Evans & Mangelsdorf, 2014). To uncover the excellent adaptability to the hygrothermal environment in Hu sheep, the regulatory mechanisms of methylation under stress remain to be further investigated in next step.

## FUNDING INFORMATION

This work was supported by the Special Financial Fund of ChongQing (grand number: 22544C: 23522C: 23511C).

## CONFLICT OF INTEREST STATEMENT

The authors declare no conflict of interest.

## ETHICS STATEMENT

The research was prospectively reviewed and approved by a duly constituted ethics committee. All the procedures involved in handling and collecting the samples from sheep were approved by the ethics committee of the Chongqing Academy of animal sciences (Approval Number: xky‐20,240,311).

## Supporting information


Figure S1.

File S1.

File S2.

File S3.

File S4.

File S5.

File S6.

## Data Availability

The raw sequence data reported in this paper have been deposited in the Genome sequence Archive in National Genomics Data Center for Bioinformation/Beijing Institute of Genomics, Chinese Academy of Sciences (GSA:CRA012705) that are publicly accessible at https://ngdc.cncb.cn/gsa.
