# Supplementary figures and images for: The epigenetic mechanisms of adaption to the hot and humid climate in Hu sheep (Ovis aries)
Source: Physiol Rep. 2024 Dec 26;12(24):e16164. doi: 10.14814/phy2.16164 (PMC11671241; doi:10.14814/phy2.16164)

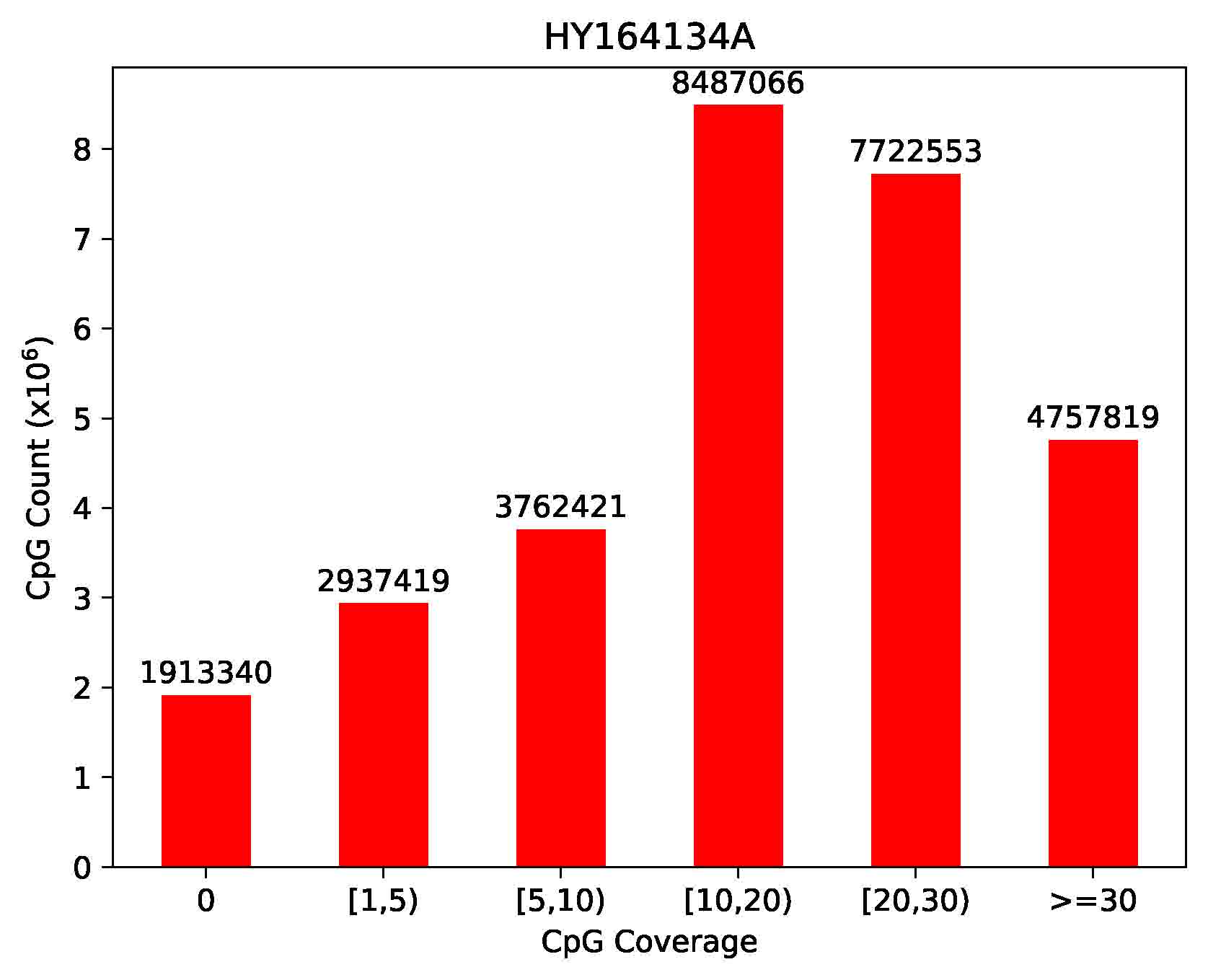

Supplement: Supplementary file 1 — Figure S1. [file PHY2-12-e16164-s001.zip › PHYSREP-2023-10-444-f08-z-.jpg]

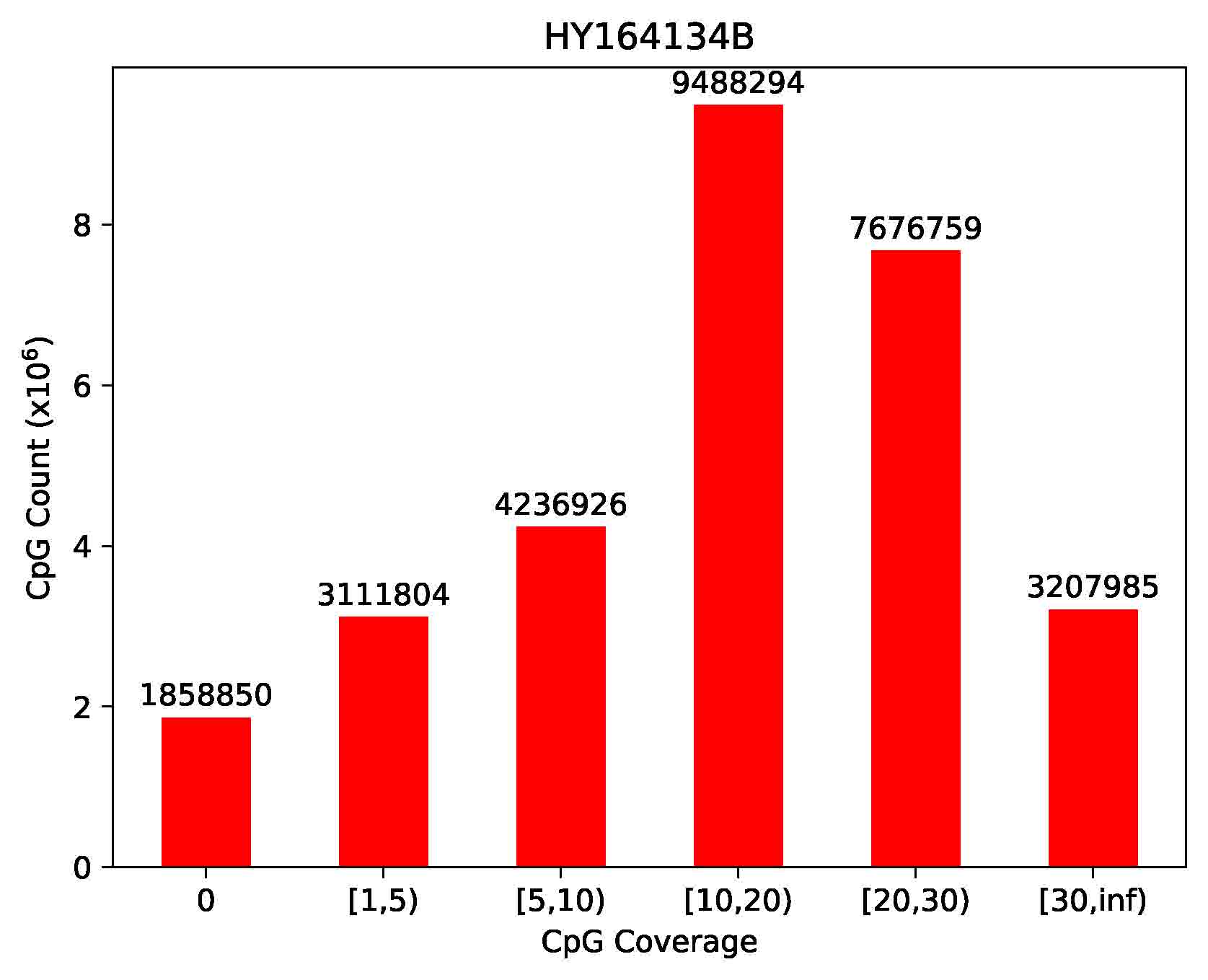

Supplement: Supplementary file 1 — Figure S1. [file PHY2-12-e16164-s001.zip › PHYSREP-2023-10-444-f09-z-.jpg]

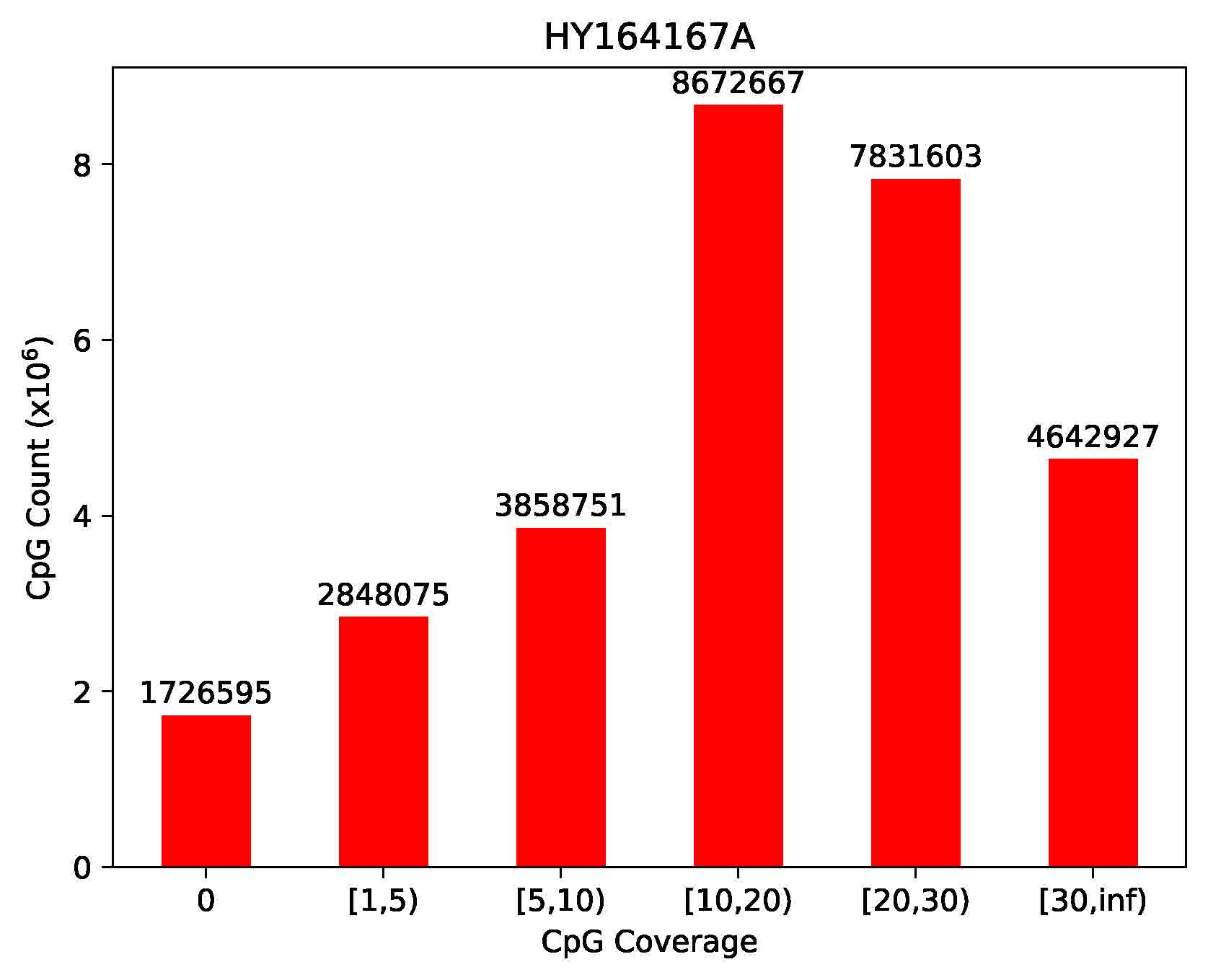

Supplement: Supplementary file 1 — Figure S1. [file PHY2-12-e16164-s001.zip › PHYSREP-2023-10-444-f10-z-.jpg]

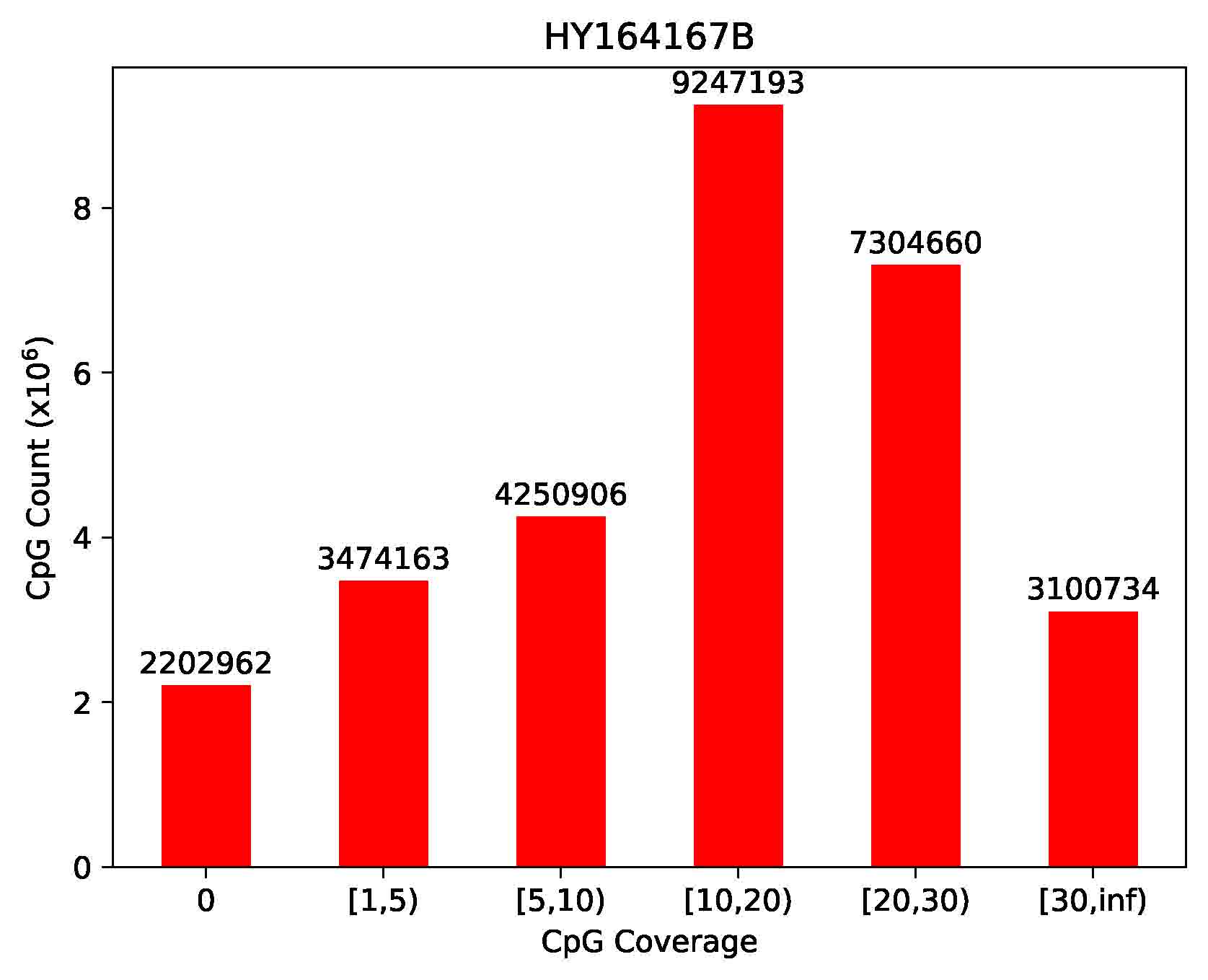

Supplement: Supplementary file 1 — Figure S1. [file PHY2-12-e16164-s001.zip › PHYSREP-2023-10-444-f11-z-.jpg]

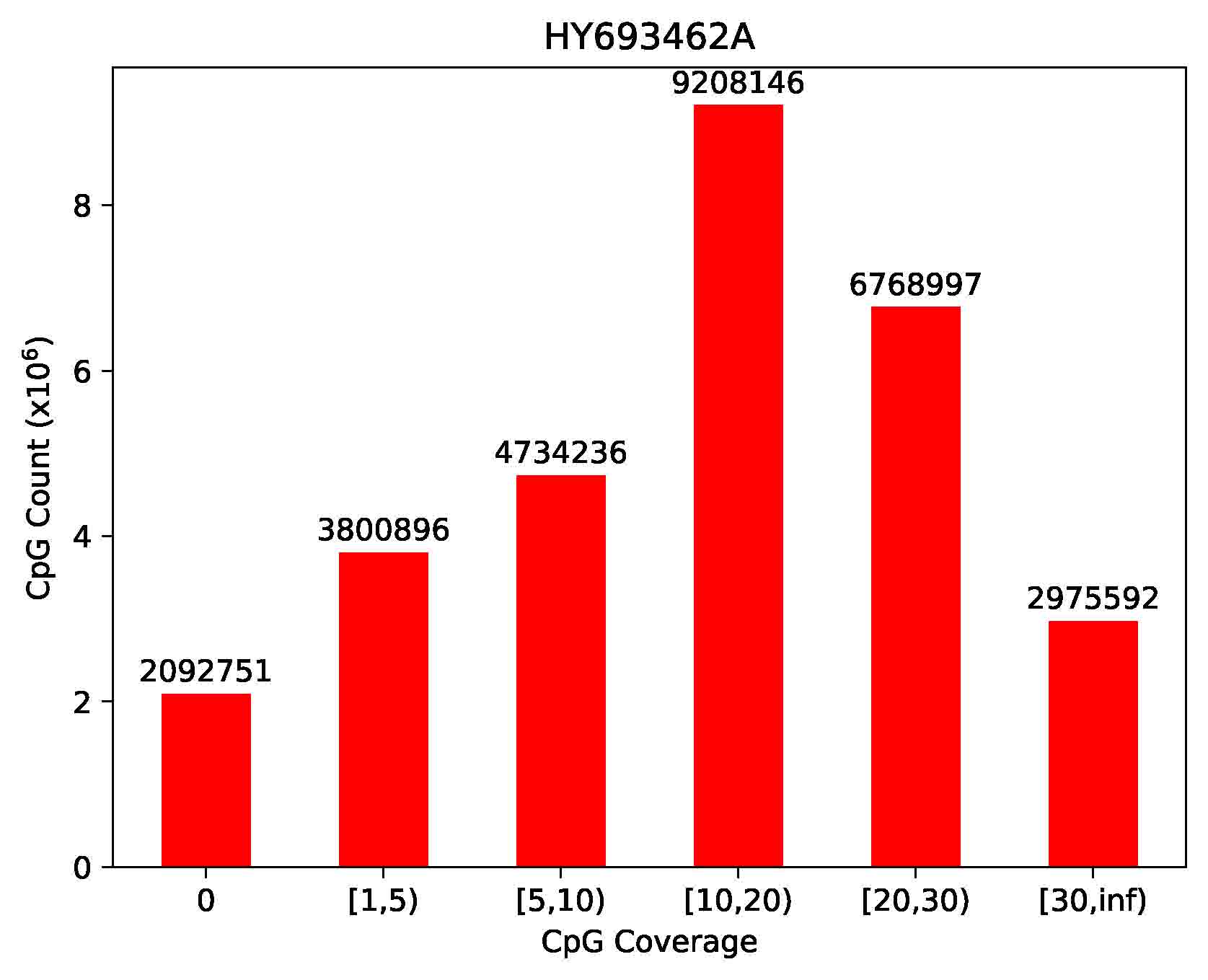

Supplement: Supplementary file 1 — Figure S1. [file PHY2-12-e16164-s001.zip › PHYSREP-2023-10-444-f12-z-.jpg]

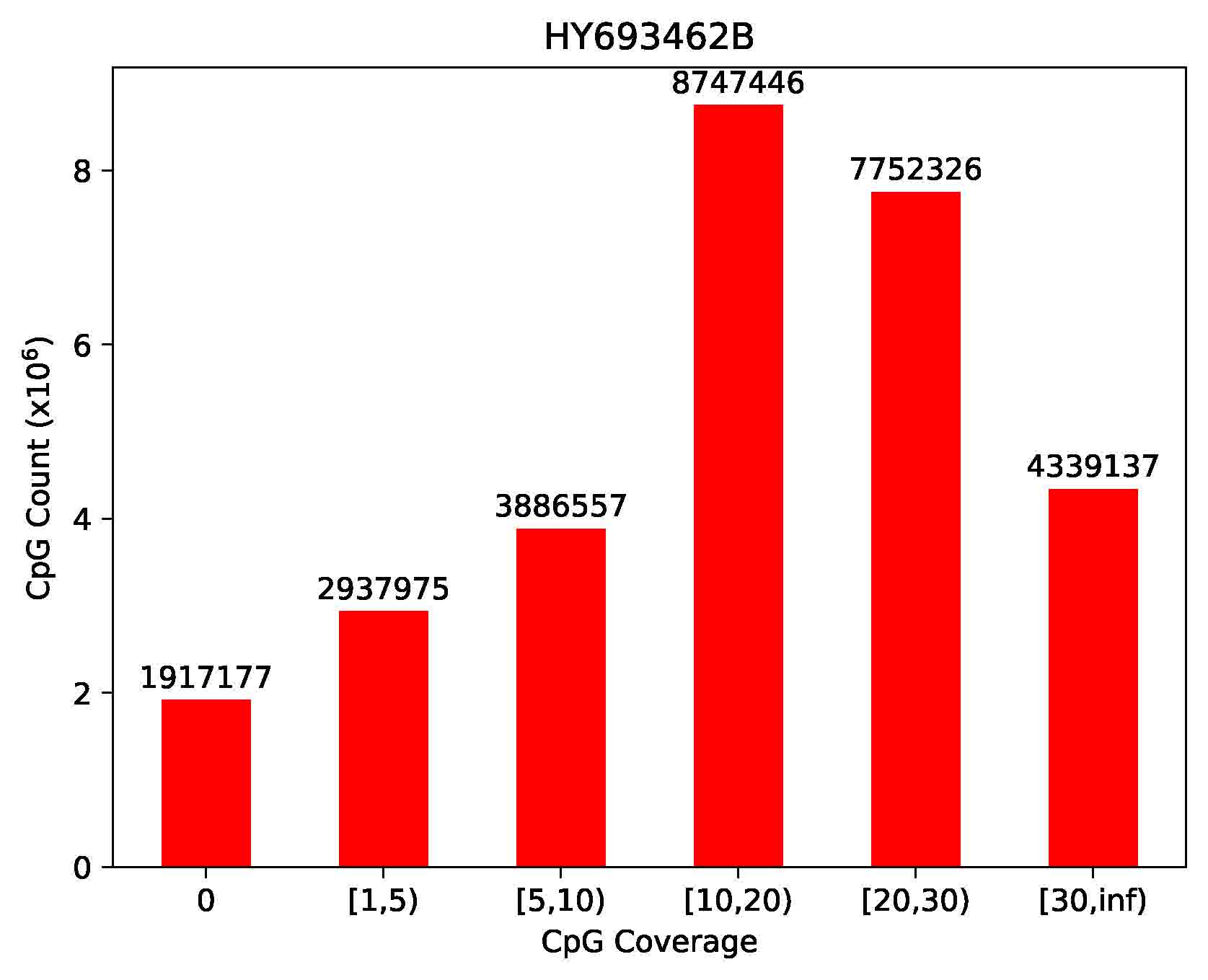

Supplement: Supplementary file 1 — Figure S1. [file PHY2-12-e16164-s001.zip › PHYSREP-2023-10-444-f13-z-.jpg]

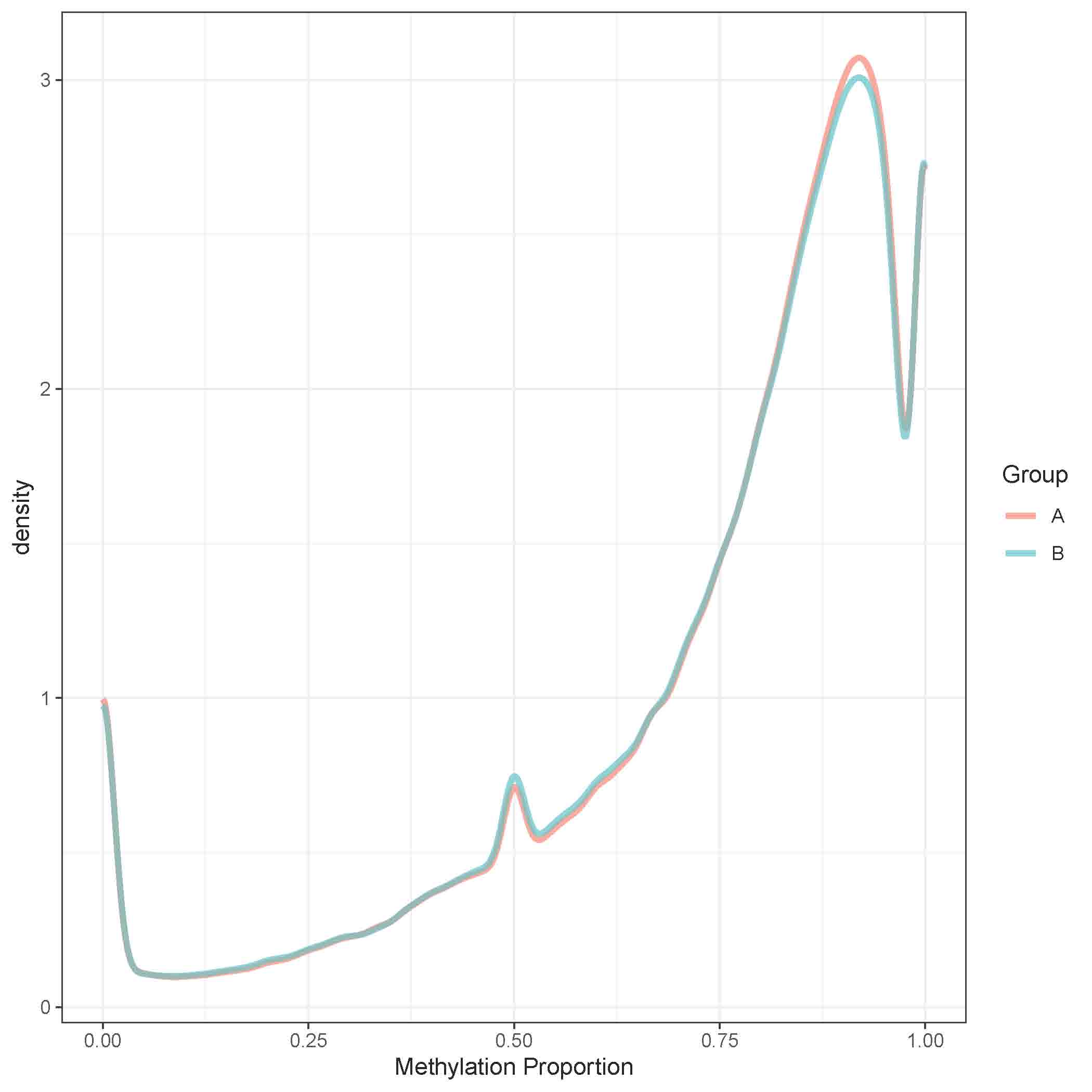

Supplement: Supplementary file 1 — Figure S1. [file PHY2-12-e16164-s001.zip › PHYSREP-2023-10-444-f14-z-.jpg]

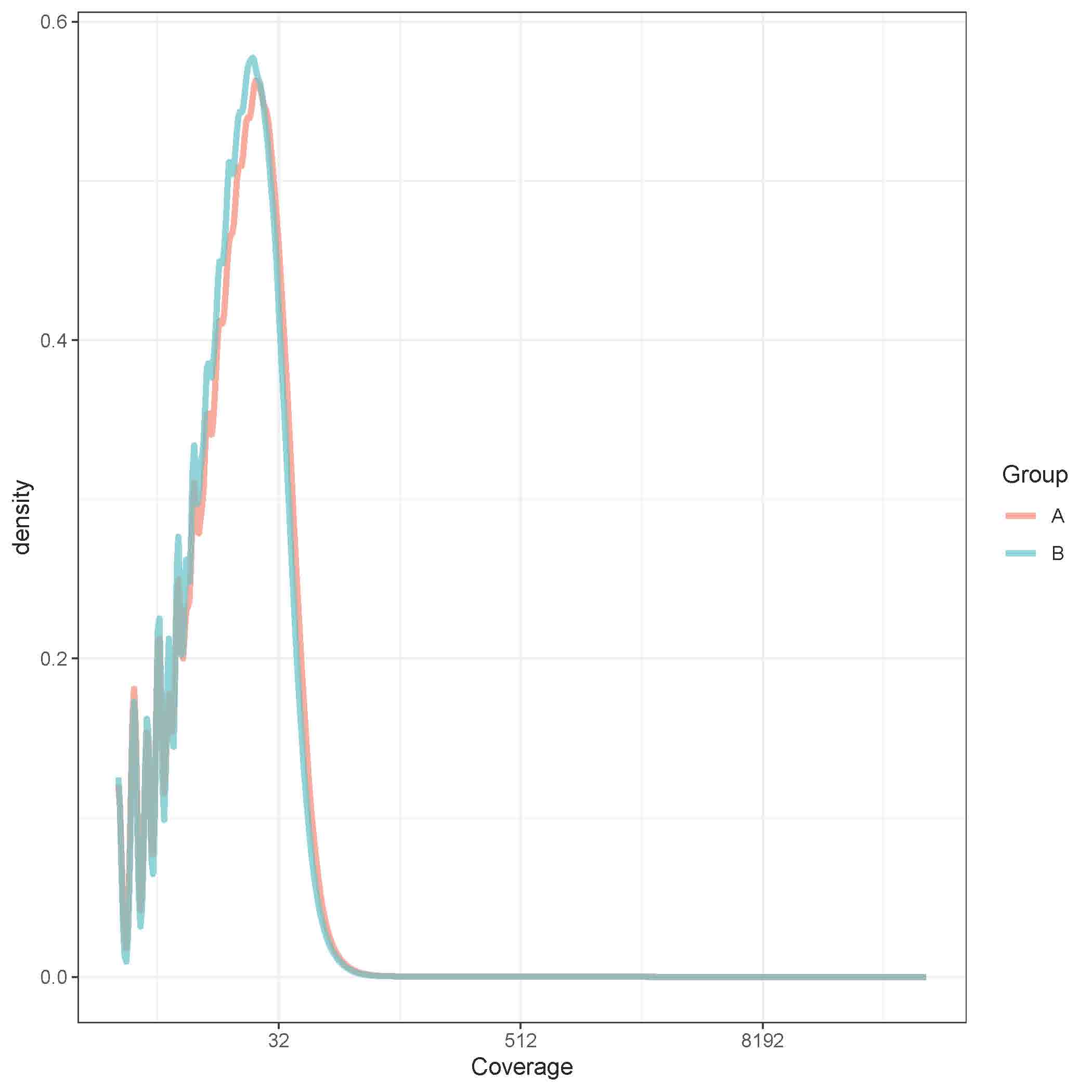

Supplement: Supplementary file 1 — Figure S1. [file PHY2-12-e16164-s001.zip › PHYSREP-2023-10-444-f15-z-.jpg]

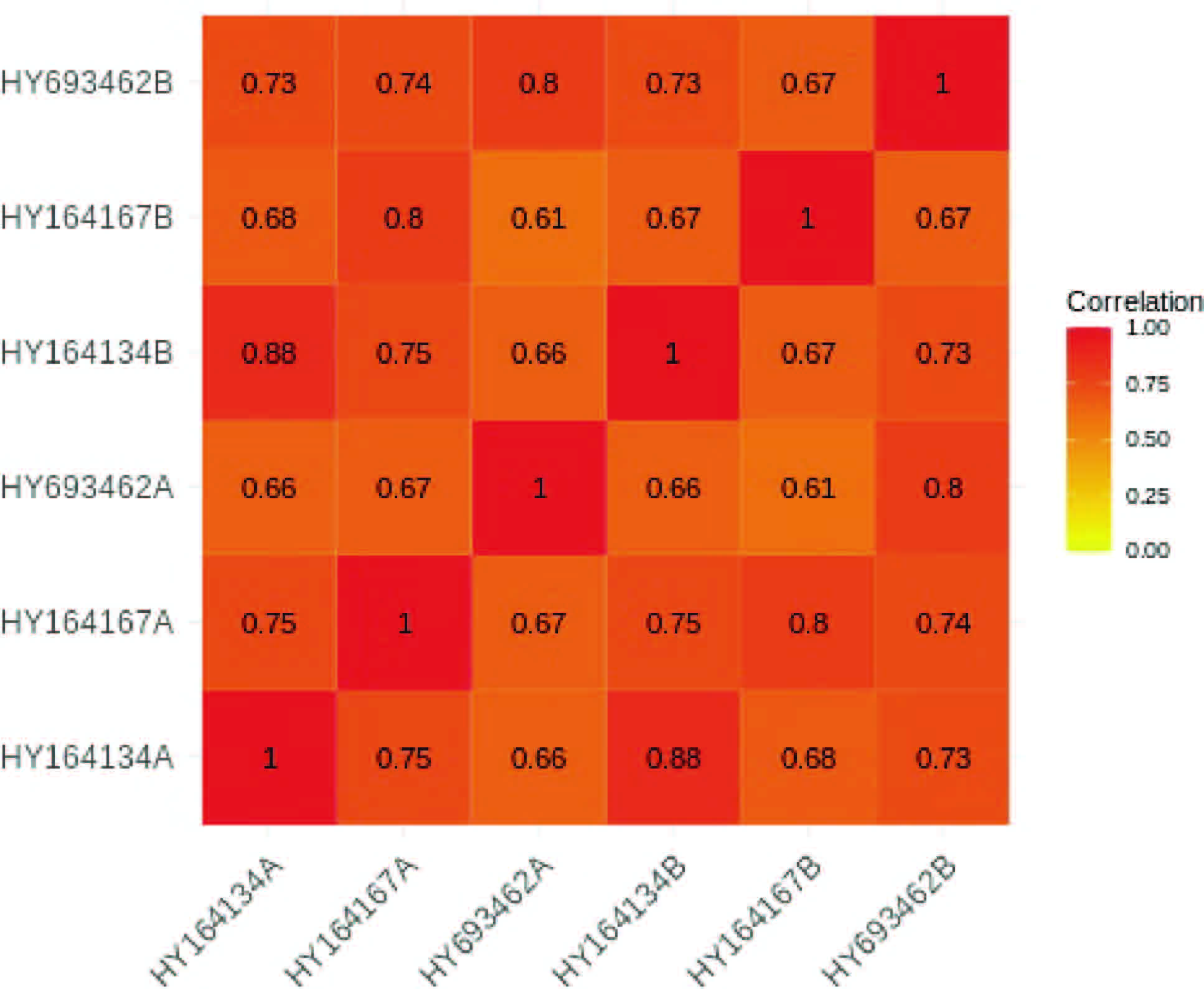

Supplement: Supplementary file 1 — Figure S1. [file PHY2-12-e16164-s001.zip › PHYSREP-2023-10-444-f16-z-.jpg]

# HY164134A

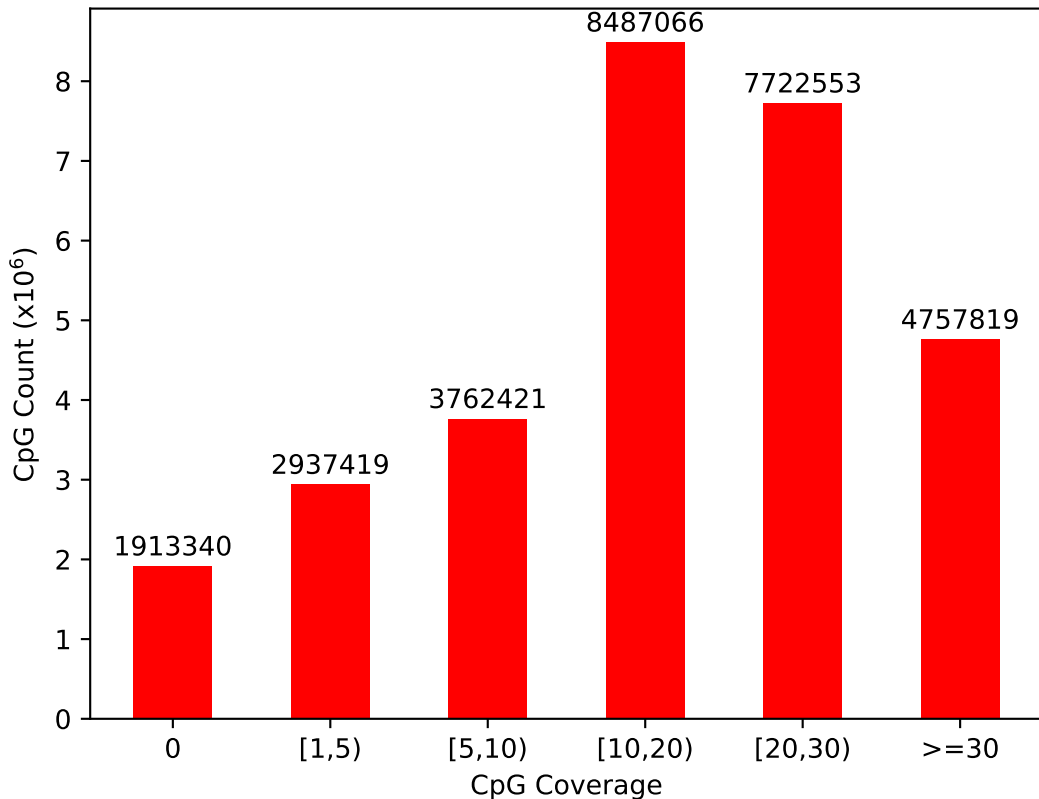

# HY164134B

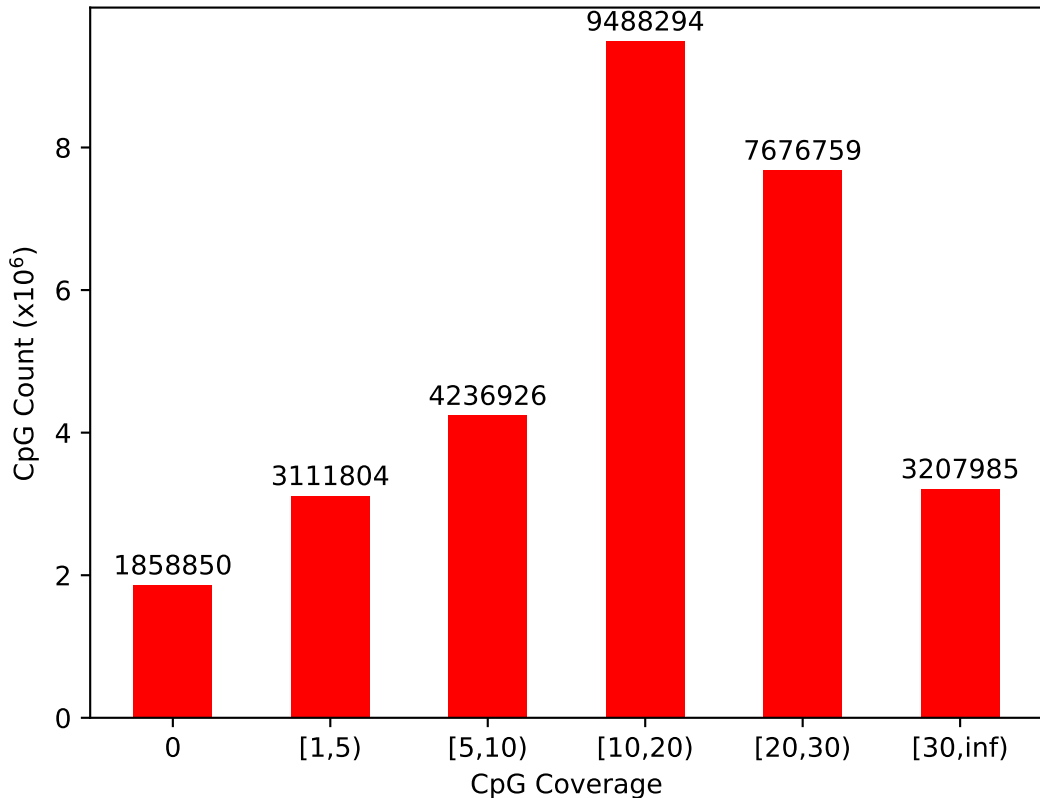

# HY164167A

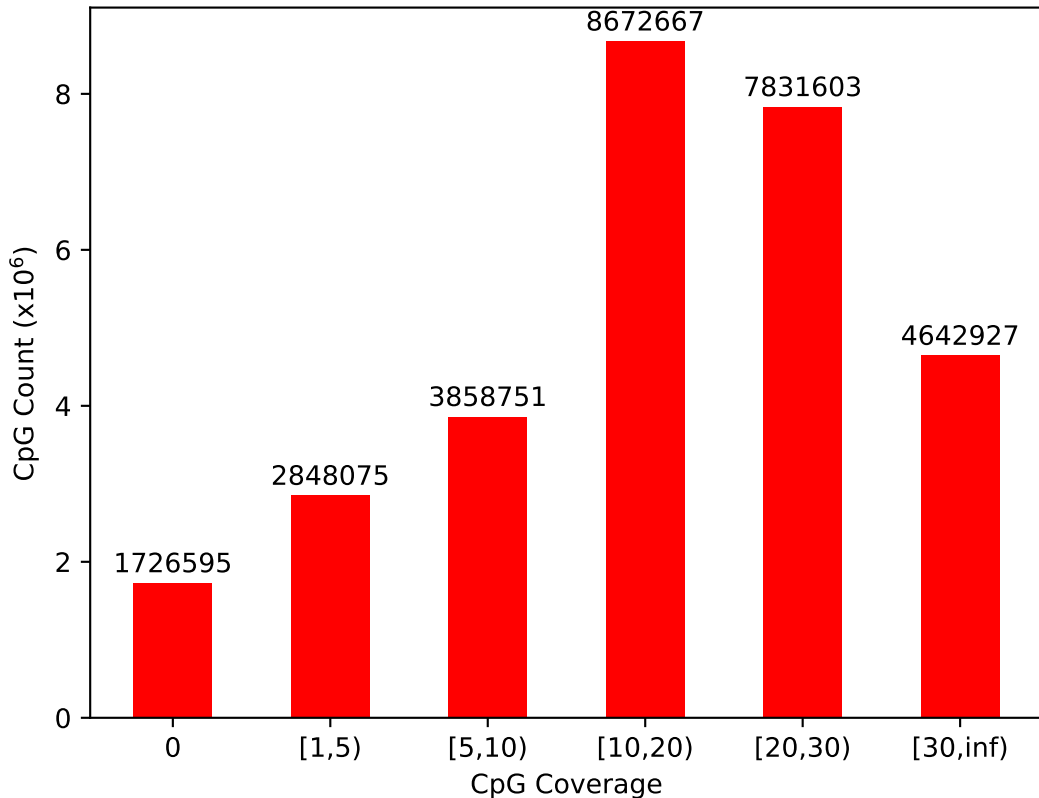

# HY164167B

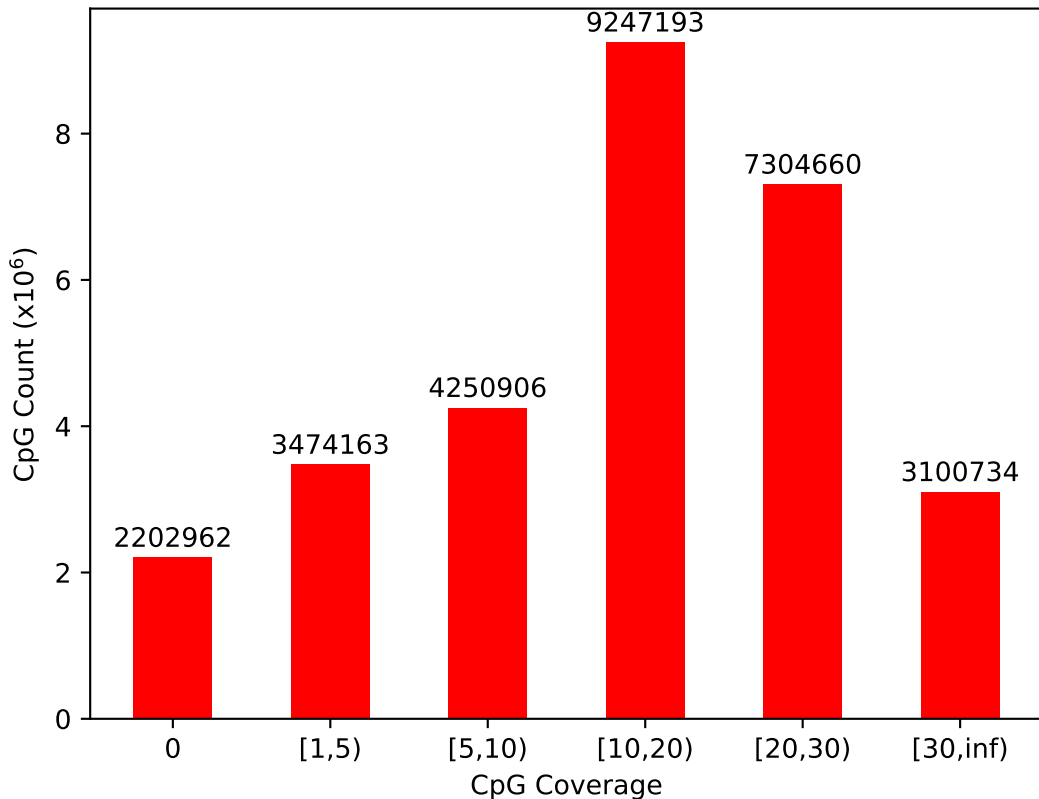

# HY693462A

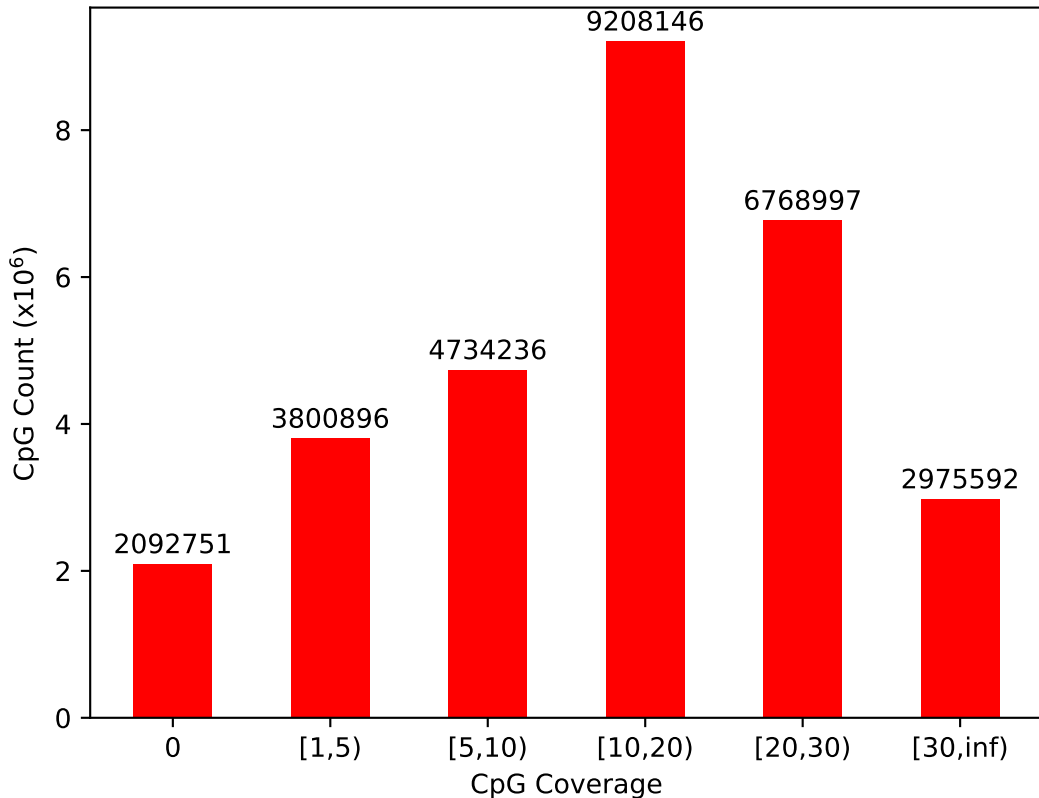

# HY693462B

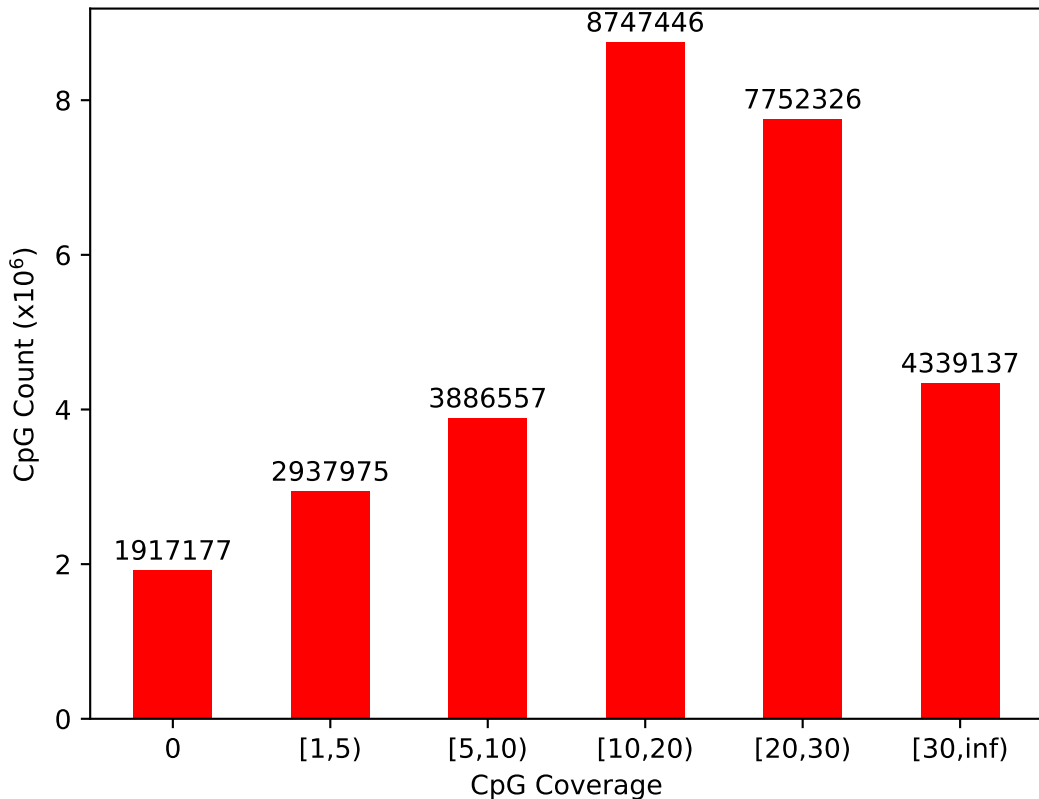

Supplement: Supplementary file 4 — File S3. [file PHY2-12-e16164-s006.pdf]

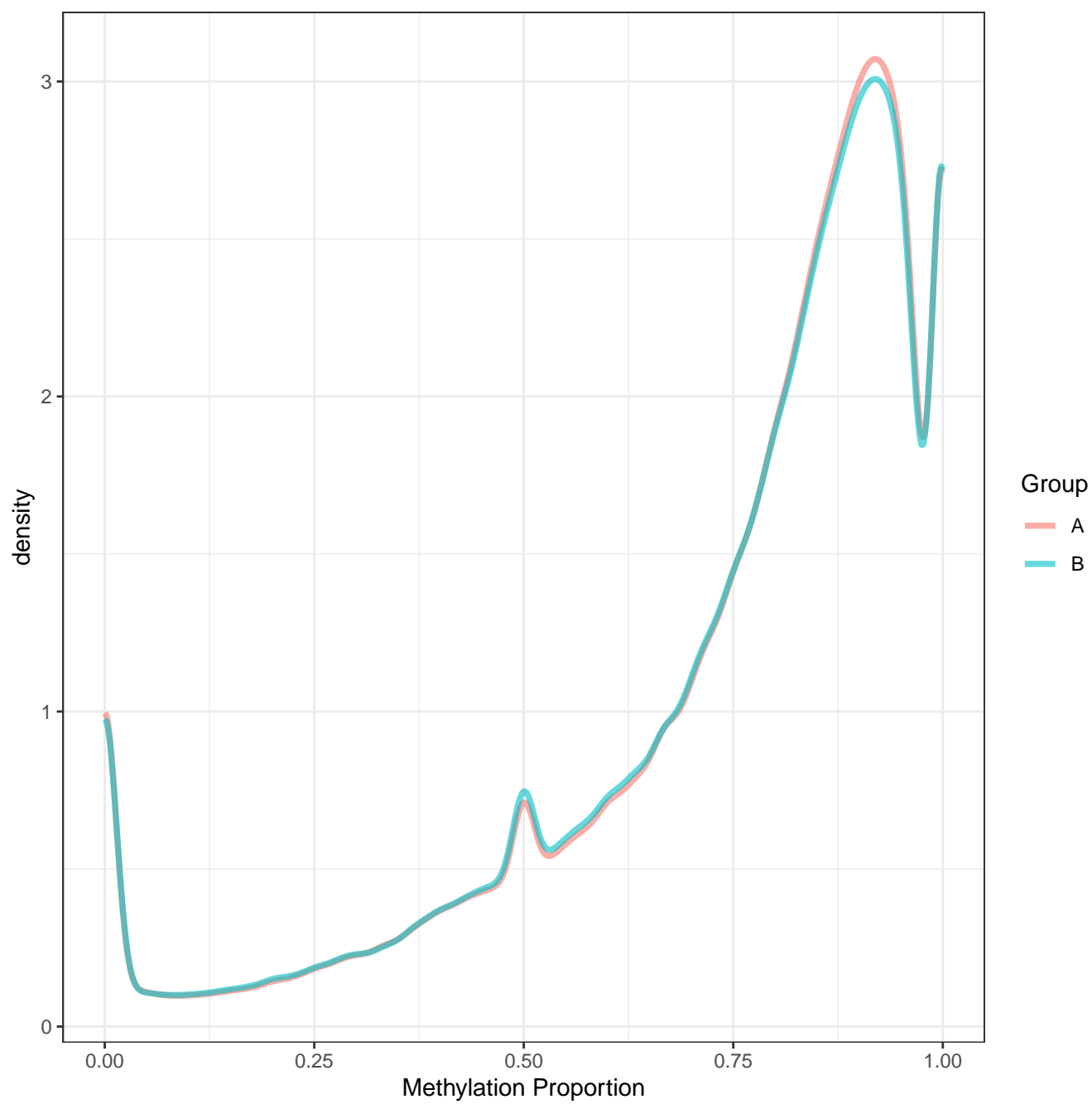

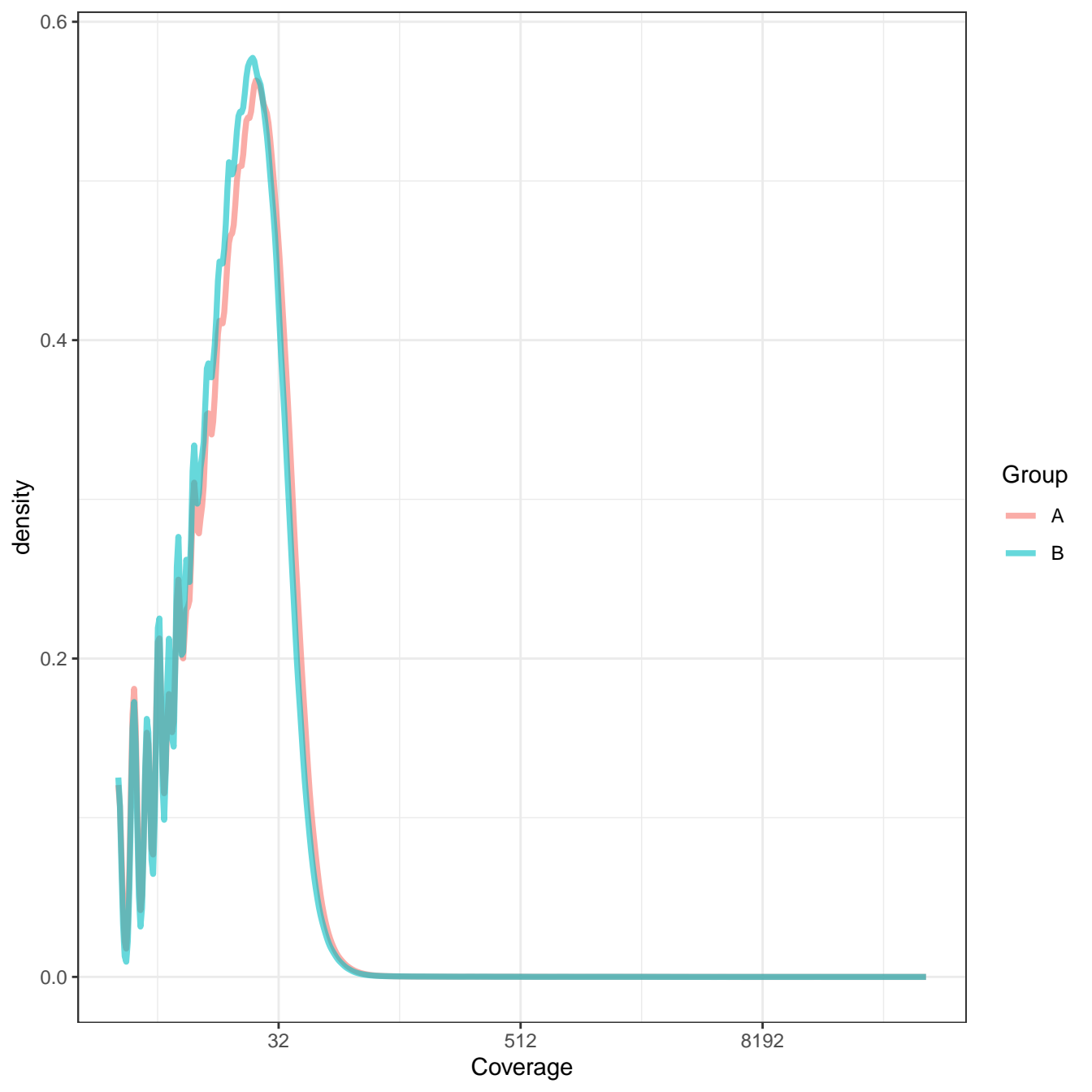

Supplement: Supplementary file 5 — File S4. [file PHY2-12-e16164-s004.pdf]

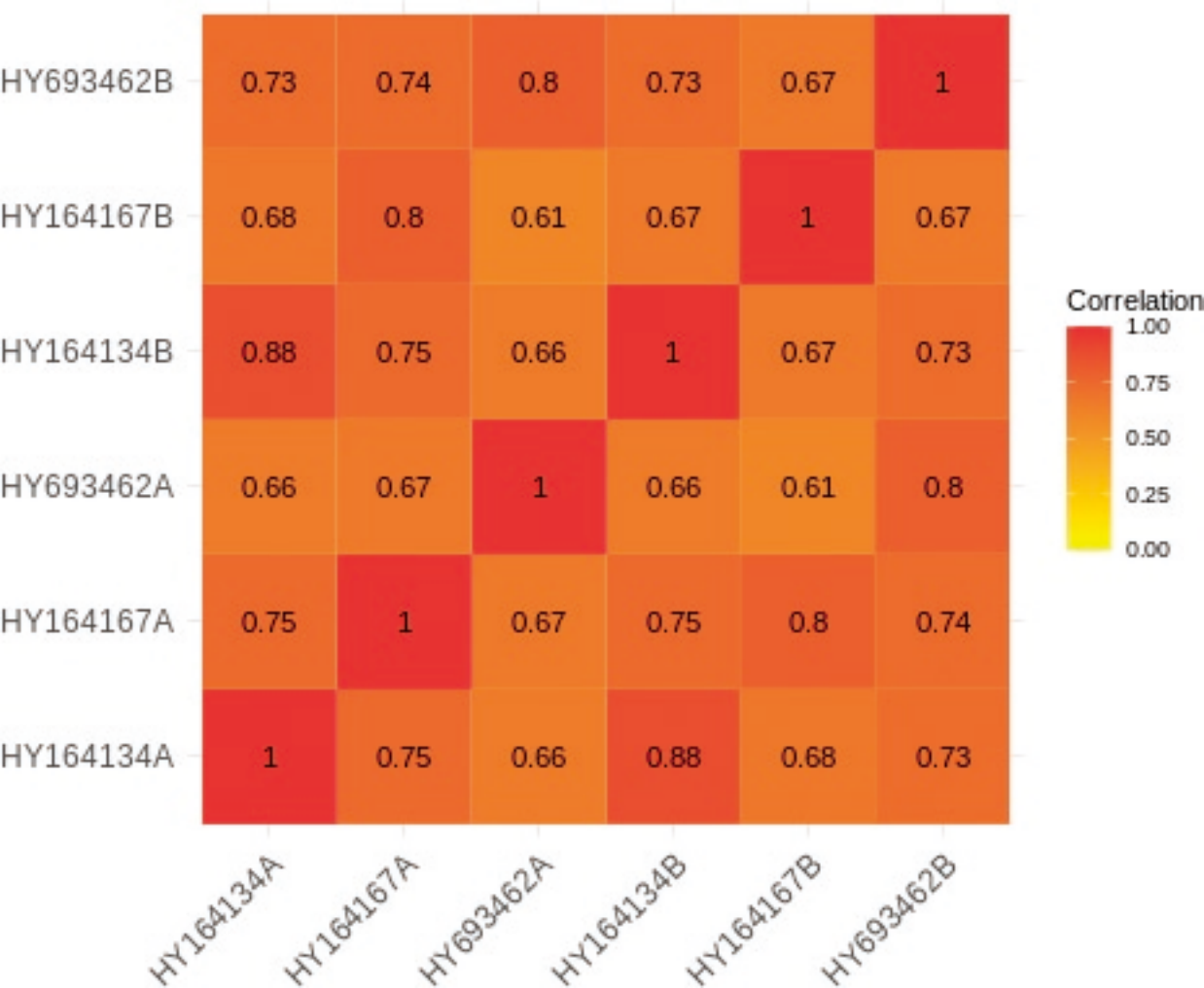

Supplement: Supplementary file 6 — File S5. [file PHY2-12-e16164-s007.pdf]

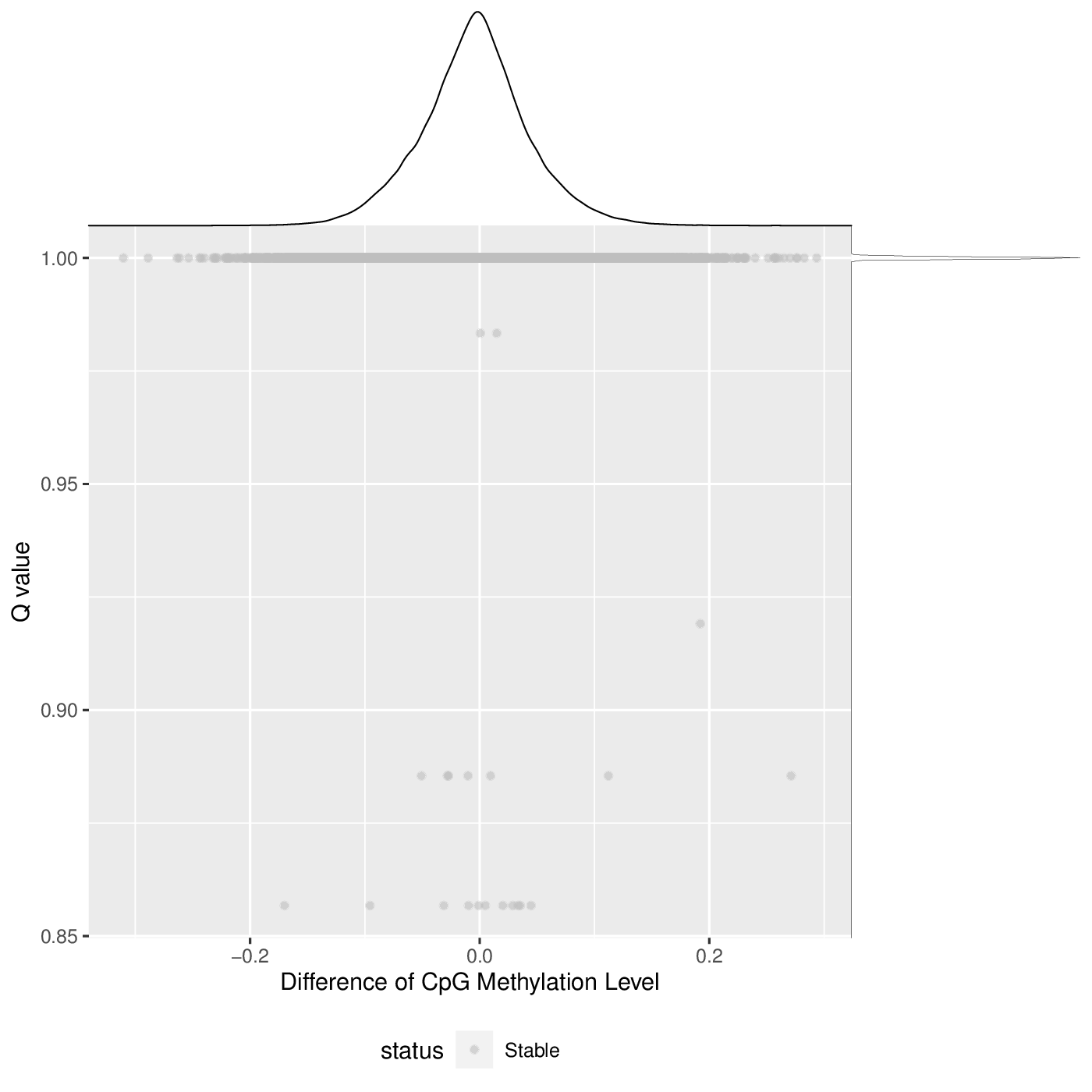

Supplement: Supplementary file 7 — File S6. [file PHY2-12-e16164-s005.png]
