# Supplementary material for: The epigenetic mechanisms of adaption to the hot and humid climate in Hu sheep (Ovis aries)
Source: Physiol Rep. 2024 Dec 26;12(24):e16164. doi: 10.14814/phy2.16164 (PMC11671241; doi:10.14814/phy2.16164)
Supplement: Supplementary file 2 — File S1. [file PHY2-12-e16164-s003.docx]

| Supplementary file 1 Overview of the dada using whole-genome methylation sequencing | | | | | |
| --- | --- | --- | --- | --- | --- |
| Sample No. | **Mapping rate** | **Proper paired reads** | **PCR repetition rate** | **Organelle contamination** | **Final available read pairs** |
| HY164134A | 84.9% | 301,739,709 | 13.45% | 0.45% | 259,965,944 |
| HY164134B | 84.8% | 260,252,734 | 13.75% | 0.44% | 223,475,876 |
| HY164167A | 85.3% | 292,954,147 | 16.94% | 0.48% | 242,159,860 |
| HY164167B | 86.7% | 266,914,183 | 15.81% | 0.43% | 223,741,762 |
| HY693462A | 86.4% | 264,012,569 | 15.54% | 0.42% | 222,042,605 |
| HY693462B | 85.7% | 298,859,728 | 18.29% | 0.54% | 242,862,419 |
