# Supplementary material for: The epigenetic mechanisms of adaption to the hot and humid climate in Hu sheep (Ovis aries)
Source: Physiol Rep. 2024 Dec 26;12(24):e16164. doi: 10.14814/phy2.16164 (PMC11671241; doi:10.14814/phy2.16164)
Supplement: Supplementary file 3 — File S2. [file PHY2-12-e16164-s002.docx]

| Supplementary file 2 The statistics of CG site and the average methylation levels of CG, CHG and CHH in the each sample | | | | | | | | |
| --- | --- | --- | --- | --- | --- | --- | --- | --- |
| Sample | **CG count of in whole genome** | **CG count in individual sample** | **Ratio of individual to total** | **Mean depth of CG** | **Mean methylation level of CG** | **Mean methylation level of CHG** | **Mean methylation level of CHH** | **Conversion rate of Bisulfite** |
| HY164134A | 29,580,618 | 27,667,278 | 93.53% | 18.93 | 72.23% | 0.32% | 0.29% | 99.79% |
| HY164134B | 29,580,618 | 27,721,768 | 93.72% | 17.32 | 72.19% | 0.36% | 0.32% | 99.75% |
| HY164167A | 29,580,618 | 27,854,023 | 94.16% | 18.88 | 72.48% | 0.39% | 0.35% | 99.71% |
| HY164167B | 29,580,618 | 27,377,656 | 92.55% | 16.94 | 73.10% | 0.34% | 0.31% | 99.78% |
| HY693462A | 29,580,618 | 27,487,867 | 92.93% | 16.35 | 73.61% | 0.37% | 0.33% | 99.74% |
| HY693462B | 29,580,618 | 27,663,441 | 93.52% | 18.53 | 72.83% | 0.34% | 0.31% | 99.78% |
